# Effects of Essential Oils-Based Supplement and *Salmonella* Infection on Gene Expression, Blood Parameters, Cecal Microbiome, and Egg Production in Laying Hens

**DOI:** 10.3390/ani11020360

**Published:** 2021-02-01

**Authors:** Georgi Yu. Laptev, Elena A. Yildirim, Larisa A. Ilina, Valentina A. Filippova, Ivan I. Kochish, Elena P. Gorfunkel, Andrei V. Dubrovin, Evgeni A. Brazhnik, Valeriy G. Narushin, Natalia I. Novikova, Oksana B. Novikova, Timur P. Dunyashev, Vladimir I. Smolensky, Peter F. Surai, Darren K. Griffin, Michael N. Romanov

**Affiliations:** 1 BIOTROF+ Ltd., 8 Malinovskaya St, liter A, 7-N, Pushkin, 196602 St Petersburg, Russia; georg-laptev@rambler.ru (G.Y.L.); deniz@biotrof.ru (E.A.Y.); ilina@biotrof.ru (L.A.I.); filippova@biotrof.ru (V.A.F.); alenkafev@mail.ru (E.P.G.); dubrowin.a.v@yandex.ru (A.V.D.); bea@biotrof.ru (E.A.B.); natalia-iv-nov@rambler.ru (N.I.N.); timur@biotrof.ru (T.P.D.); 2 K. I. Skryabin Moscow State Academy of Veterinary Medicine and Biotechnology, 23 Akademika Skryabina St, 109472 Moscow, Russia; kochish.i@mail.ru (I.I.K.); smolensky-vgnki@mail.ru (V.I.S.); m.romanov@kent.ac.uk (M.N.R.); 3 Research Institute for Environment Treatment, 69032 Zaporozhye, Ukraine; val@vitamarket.com.ua; 4 Vita-Market Ltd., Yuzhnoye Shosse 1, 69032 Zaporozhye, Ukraine; 5 All-Russian Veterinary Research Institute of Poultry Science—Branch of the Federal State Budget Scientific Institution Federal Scientific Centre “All-Russian Poultry Research and Technological Institute” of the Russian Academy of Sciences, 48 Chernikova St, Lomonosov, 198412 St Petersburg, Russia; ksuvet@mail.ru; 6 Department of Microbiology and Biochemistry, Faculty of Veterinary Medicine, Trakia University, 6000 Stara Zagora, Bulgaria; 7 Department of Animal Nutrition, Faculty of Agricultural and Environmental Sciences, Szent Istvan University, H-2103 Gödöllo, Hungary; 8 School of Biosciences, University of Kent, Canterbury, Kent CT2 7NJ, UK; D.K.Griffin@kent.ac.uk

**Keywords:** essential oils-based phytobiotic, *Salmonella enterica* serovar Enteritidis, gene expression, cecum microbiome, blood biochemical/immunological parameters, laying hens

## Abstract

**Simple Summary:**

Salmonellosis is one of the most severe zoonotic diseases transmitted to humans through animal products (especially poultry meat and eggs). Essential oils (EOs)-based feed additives in poultry nutrition are a possible alternative replacement of antimicrobials to fight this infection. In the present study on laying hens, we tested a phytobiotic, Intebio^®^, and elucidated formation of immune response and changes in cecal microbiocenosis and biochemical/immunological variables in blood caused by *Salmonella*. Changes in differential gene expression were observed at both one and seven days post-inoculation in the hens’ intestines, revealing similarities with known mammalian/human tissue-specific expression. The results of this study suggest that the challenge of birds with *Salmonella* had a negative effect, while phytobiotic intake had a positive effect on the status of their gastrointestinal microbiome, their level of metabolism, and their performance.

**Abstract:**

One of the main roles in poultry resistance to infections caused by *Salmonella* is attributed to host immunity and intestinal microbiota. We conducted an experiment that involved challenging Lohmann White laying hens with *Salmonella* Enteritidis (SE), feeding them a diet supplemented with an EOs-based phytobiotic Intebio^®^. At 1 and 7 days post-inoculation, the expression profiles of eight genes related to immunity, transport of nutrients in the intestine, and metabolism were examined. Cecal microbiome composition and blood biochemical/immunological indices were also explored and egg production traits recorded. As a result, the SE challenge of laying hens and Intebio^®^ administration had either a suppressive or activating effect on the expression level of the studied genes (e.g., *IL6* and *BPIFB3*), the latter echoing mammalian/human tissue-specific expression. There were also effects of the pathogen challenge and phytobiotic intake on the cecal microbiome profiles and blood biochemical/immunological parameters, including those reflecting the activity of the birds’ immune systems (e.g., serum bactericidal activity, β-lysine content, and immunoglobulin levels). Significant differences between control and experimental subgroups in egg performance traits (i.e., egg weight/number/mass) were also found. The phytobiotic administration suggested a positive effect on the welfare and productivity of poultry.

## 1. Introduction

Contamination with *Salmonella* affects an estimate of 20% of poultry meat [1]. This pathogen has the ability to remain viable for considerable periods of time in various biological and nonbiological habitats due to the formation of biofilms. *Salmonella* spp. are widely represented in the environment, and the intestinal tract of animals and birds is where they are found most [2,3]. *Salmonella enterica* can cause severe zoonotic disease and is transmitted to humans via raw animal products, including poultry meat and eggs [4,5].

Intestinal microbiota plays one of the dominant roles in the resistance of chickens to *Salmonella* infections [6]. Its composition correlates to a large extent with the responsiveness to gastrointestinal diseases caused by pathogens [7,8]. It can also reflect inflammatory processes, an influx of phagocytes and lymphocytes from the circulatory system, an increase in the amount of pathogenic microflora, and its toxin-producing activity. Symbiotic intestinal microbiota in chickens has long been considered as a separate multifunctional “organ” that is very important in the functioning of the whole organism, influencing health and performance [9]. Birds have such a peculiar feature of digestive system as the lowest rate of passage of the chyme in cecum, compared with the other intestinal parts, which creates an ideal environment for the propagation of various microorganisms.

Disturbance in the composition of the intestinal microflora in birds due to antibiotic therapy significantly increases their susceptibility to *S. enterica* [10,11]. This highlights the need to search for and develop feed additive alternatives to antibiotics. Apart from the use of antibiotics, an important way to prevent bacterial diseases is to modulate the intestinal protection using feed ingredients or prebiotics [12,13,14]. In recent years, evidence has accumulated that essential oils (EOs) can be effective natural immunomodulators [15,16,17]. Plant EOs are terpenes and terpenoids, as well as aromatic and aliphatic compounds [18]. Some phenolic compounds and terpenes have been reported to have anti-inflammatory activity [19,20]. The antimicrobial property of several plant-derived EOs has been demonstrated against gram-negative and gram-positive bacteria [21], including *S. enterica* serovar Enteritidis (SE) [22,23]. It was also noted that the addition of phytochemicals to feed had a positive effect on the secretion of pancreatic and intestinal enzymes in broilers [24]. It promoted nutrient absorption and improved feed conversion in poultry [25]. Many ingredients in the composition of EOs are generally recognized as safe (GRAS) compounds permitted by the U.S. Food and Drugs Administration when used for feed purposes [26].

When intestinal pathogens, including SE, enter the host organism, they are able to modulate the hosts’ transcriptional program [27,28]. Recognition of a pathogen is a complex process, triggers subsequent changes in the expression levels of many host genes, and depends on a number of factors, such as the host genotype, its immunological status, dietary factors, etc.

The epithelial membrane in the intestines not only protects the host from pathogens present in the intestinal lumen but, also, performs the function of absorbing nutrients through numerous ion channels and transporters present on the apical intestinal epithelial border. Microbial infections can affect the transport of ions in the intestines. This depends on such factors as host immunity, virulence of pathogenic microorganisms, structural organization of the mucous membrane of a particular segment of the intestines, and age of the animal [6,29,30]. Due to infection with pathogens, the functional disruption of ion transporters, such as cystic fibrosis transmembrane conductance regulator (CFTR) and epithelial Na^+^/H^+^ exchanger SLC9A3 [29,30], can result in diarrhea, malabsorption, and inflammation of the intestine [31]. This leads to lower efficiency in the production of poultry produce.

In chickens, *Salmonella* recognition in the cecum initially occurs by means of Toll-like receptors (TLR) [32]. This is followed by the subsequent induction of genes associated with the synthesis of chemokines, cytokines, and effector genes that form the basis of the innate immune system. It leads to the infiltration of heterophiles, macrophages, and B and T lymphocytes [33,34]. It also has been found in chickens that such cytokines as interleukin-1β (IL1B), interleukin-6 (IL6), interleukin-17 (IL17A), interleukin-22 (IL22), and others contribute to the development of the inflammatory response. The main function of other peptides, chemokines, is to regulate the movement of leukocytes. In the chicken genome, a number of genes associated with chemokine synthesis have been identified, one of them being *IL8L1* (*K60* or *CXCLi1*), involved in signaling between immune cells [35].

Antimicrobial peptides called defensins also contribute to counteracting infections caused by *Salmonella* spp. [36,37]. They embrace three main groups of α-, β-, and θ-defensins [38,39,40]. While α-defensins were found only in mammals [38] and θ-defensins in all vertebrates [41], β-defensins (or gallinacins), such as AvBD1 (Gal-1 and Gal-1α), AvBD2 (Gal-2), AvBD4 (Gal-4), AvBD10 (Gal-10), and others [41,42,43,44], are specific to birds, including chickens. These antimicrobial peptides influence pathogenic microbes by interacting with the negatively charged phospholipid bilayer in the cell membrane, which leads to the destruction of bacterial cells [42]. It was also suggested that β-defensins in birds exhibit a more pronounced efficacy against Gram-positive bacteria than Gram-negative bacteria because of their structural conformation [45]. The expression of genes associated with the synthesis of β-defensins has been observed in various organs and tissues in chickens, including intestinal epithelial cells [46].

To date, the available information about the immune reaction in birds in response to *S. enterica*–related lesions is contradictory and does not give a clear indication as to its formation and manifestation. In addition to observations that the infection of chickens with *Salmonella* is, as a rule, characterized by the induction of an inflammatory response and upregulation of many genes, it was noted that the expression level of some other genes can be reduced due to infection with this pathogen [47]. In particular, the infection of five-month-old chickens with SE resulted at 10 days post-inoculation (dpi) in the downregulation of 32 various genes in the liver. These genes mainly belonged to two functional categories: the control of metabolic functions and the cell cycle. The greatest reduction of expression was found for some genes, including *CALB1*, associated with the synthesis of calbindin 1 [47].

The challenge of laying hens with *Salmonella* has long been shown to have a negative impact on egg production [48,49]. In addition, the effect of the pathogen on the blood parameters of birds was studied [50]; however, data on the effect of SE on the expression of immunity and productivity genes are limited. Therefore, further research is needed to explore the expression of these and other genes related to resistance, metabolism, and productivity in response to *Salmonella* infection in hens.

There are also homologous genes not only in birds but, also, in other amniotes and humans that are over- or under-expressed in the gastrointestinal tract (GIT), including the vermiform appendix and intestine [51]. These include *IL6*, *IL8*, and *CALB1*, as well as *SLC5A1* (solute carrier family 5 member 1), *CA2* (carbonic anhydrase 2), *RARRES1* (retinoic acid receptor responder 1), and *BPIFB3* (BPI fold containing family B member 3). SLC5A1 is part of the glucose (GLUC) carrier family [52]. CA2 participates in many processes, with the gene expression also found in the intestines. In addition, the chicken homolog also affects eggshell calcification [53]. RARRES1 and BPIFB3 are involved in the regulation of endopeptidase activity and innate immune response, respectively, and are also associated with the synthesis of eggshell proteins [54,55,56,57]. It would therefore be of great interest to clarify the effects of infection and phytogenic dietary supplementation on the functioning of these genes in the chicken cecum.

Important indicators characterizing the level of metabolic processes and the overall functional state of the body are certain elements and properties of the blood. Change in number of formed elements in the blood is due to certain paratypical factors whose descriptions facilitate understanding the state and dynamics of the main physiological functions of the organism, especially when it is infected with pathogenic microflora. For example, an increased activity of alanine aminotransferase (ALT) in the blood of layer chickens was previously shown to be due to infection with *Salmonella* [58,59].

When developing preventive measures to combat *Salmonella*, it should be borne in mind that the basis of the contemporary concept to fight against pathogens would be the optimization of microecological niches in ecosystems, which should take into account the principle of self-regulation [60,61]. The search for natural environmentally friendly biopreparations based on EOs that would improve the body’s resistance and restore the normal composition of the microbiome seems to be a timely objective. One of such drugs can be a newly developed phytobiotic Intebio^®^ (BIOTROF+ Ltd., Pushkin, St Petersburg, Russia), the effectiveness of which was previously shown in swine nutrition [62,63]. Recently, we also tested the efficacy of this supplement in feeding and treating Ross 308 broiler chickens [64].

Although differential gene expression (DGE) in chickens in response to infection with SE was the focus of a number of recent studies [65,66,67], it is relevant to conduct an in-depth examination of the effects of infection on a complex of characteristics, including profiles of the cecal microbiome and regulation of the genes involved in immunity, transport, and metabolism. It is also important to conduct further investigations on the dynamics of shifts in the blood biochemical/immunological parameters and, from a practical perspective, on testing the phytobiotics such as Intebio^®^ for improving the immune response and performance. This would have the ultimate aim of preventing bacterial disease in poultry.

In this regard, the aim of the present study was to examine the composition of the cecal microbiome. Specifically, the expression of genes related to immunity, transport, and metabolism; blood biochemical/immunological indices; and egg production traits in laying hens in response to the challenge with SE, as well as the administration of Intebio^®^, a relatively novel experimental supplement derived from EOs. A better understanding of the reaction of the microbial community, as well as the function of genes associated with immunity, transport, and metabolism can help in clarifying the mechanisms of the adaptive response of chickens to *Salmonella* infection. Based on the knowledge gained, it would be feasible to develop further strategies aimed at improving the functional status, nutrition, welfare, and, ultimately, performance of layer chickens, which could make an essential contribution to the improved practices of the poultry industry.

## 2. Materials and Methods

### 2.1. Experimental Design and Sampling

The experimental protocol was set up according to the European Convention for the Protection of Vertebrate Animals used for Experimental and other Scientific Purposes, ETS No 123 and was authorized by the relevant national authority. The experiment was carried out using Lohmann White hybrid laying hens kept in a vivarium of the All-Russian Veterinary Research Institute of Poultry Science (ARVRIPS). Details on the conditions of keeping and feeding of the birds, as well as their vaccinations and welfare, are provided in the Supporting Information (SI) File S1.

The beginning of the experimental period and data collection in 80 chickens corresponded to 346 days of age when birds were randomly divided into two groups of 40 animals in each group. Group I (control) received a normal diet, while birds in Group II were fed a diet supplemented with Intebio^®^ (BIOTROF+ Ltd.) [62,63] once a day (at 9 a.m.) and in amount of 90 g per ton of feed, according to the manufacturer’s instructions for preparation use. Intebio^®^ is a feed additive with the properties of phytobiotics (Technical Specification No. TU 9362-011-50932298-2011; Registration No. PVR-2-7.11/02755) that contains a carrier (wheat bran; GOST 7169-2017), on which a mixture of EOs (derived from garlic, lemon, thyme, and eucalyptus) was applied.

At the age of 367 days, each group of birds was randomly divided into two equal subgroups, and half of them were challenged with an epizootic SE strain, so that four subgroups of 20 hens each were formed as follows: S-I, negative control subgroup fed normal diet and not challenged, S-II, control + SE challenge, S-III, Intebio^®^ intake, and S-IV, Intebio intake + SE challenge (SI File S1).

In the experiment, the strain SE 25–87 was used that was obtained from the ARVRIPS collection and isolated in 2012 from the chicken cecum. To extract the strain isolate, a sample of the chicken cecal contents was placed in a sterile physiological solution at the ratio 1:1000 and homogenized, and 3 to 4 drops of the solution were then added to a tube with a culture medium. Incubation was conducted for 24 h at 35 °C. For the strain culturing, the Rappaport medium (*Salmonella* enrichment broth; Merck KGaA, Darmstadt, Germany) was used. The medium composition included the following components (in g/l): casein peptone, 5.0, sodium chloride, 8.0, disubstituted potassium phosphate, 0.8, hexavalent magnesium chloride, 40.0, and malachite green, 0.12. Prior to culturing, 54 g of Rappaport dry magnesium medium was dissolved in 1 liter of distilled water and autoclaved for 20 min at 115 °C to ensure that the cooled medium had pH = 6.0 ± 0.2 at 25 °C. Taxonomic affiliation of the SE bacterium strain was verified using cultural morphological tests following the appropriate guidelines for the salmonellosis diagnosis procedure [68].

Challenge of the hens in S-II and S-IV was executed at 10 a.m. with the liquid culture of the SE strain in the amount of 8.69 lg colony-forming units (CFU) intramuscularly in the pectoral muscle using the standard technique [69] in order to trigger an immune response as ordinarily expected by an intramuscular vaccination. Prior to challenge, the bacterial strain was cultured overnight in the Rappaport medium at 35 °C. Nonchallenged chickens of S-I and S-III were injected with a similar volume of saline.

Presence of the inflammatory process in birds when infected with a pathogen was considered unambiguous if plasma concentrations of nitrite and N-nitroso compounds were more than 100 nM. An enzyme sensor was used to analyze nitrite and nitroso compounds in the blood of birds [69]. Additionally, to confirm the challenge of chickens with *Salmonella*, the pathological material of the intestines was plated on Modified Semisolid Rappaport-Vassiliadis Agar (Biokar Diagnostic, Allonne, France), cultured, and then analyzed using a rapid latex agglutination test (Salmonella Test Kit, Oxoid, Basingstoke, UK).

Challenged hens of S-II and S-IV were housed in separate boxes (as described in SI File S1). In the course of the experiment, the number of laid eggs and egg weight were recorded daily in the four subgroups over 27 days. Afterwards, values of the following three egg production traits were calculated in each subgroup: mean egg weight, *w*, total number of laid eggs, *N*, and egg mass, *W*, determined as the product of *W* = *wN* and equal to the total weight of laid eggs in a subgroup.

Samples for molecular biological studies and evaluation of the blood biochemical/immunological parameters were taken from randomly selected birds in the four subgroups at two experiment time points: 1 and 7 dpi. Sampling was performed under the condition of strict adherence to sterility, and euthanasia of birds was performed directly prior to obtaining samples for molecular biological studies. Samples from challenged and nonchallenged hens were collected in two different isolated laboratory rooms. The number of replications was three birds in each subgroup and on each day of sampling.

Chicken cecal tissues were sampled for analyzing DGE, and cecum contents were collected for microbiome examination. To determine blood biochemical/immunological indices, blood was taken from the wing vein of the chickens.

### 2.2. Gene Expression Analysis

DGE was assessed at 1 and 7 dpi by quantitative RT-PCR, its preliminary step being RNA extraction. Total RNA was isolated from samples using the Aurum™ Total RNA Mini Kit (Bio-Rad, Hercules, CA, USA). The tissue was ground by mixing with liquid nitrogen and homogenized, followed by the steps as prescribed in the manufacturer’s instructions. Reverse transcription reaction was conducted to produce cDNA using RNA template and the iScript™ Reverse Transcription Supermix (Bio-Rad) [70].

To analyze DGE, specific gene primers were selected for the following eight genes: *AvBD10*, *IL6*, *IL8L1*, *SLC5A1*, *CA2*, *CALB1*, *RARRES1*, and *BPIFB3* (see their details and primers in SI Table S1). Amplification reactions were performed using the SsoAdvanced™ Universal SYBR^®^ Green Supermix (Bio-Rad) following the manufacturer’s protocols (as outlined in [71]). Estimation of the relative DGE was done using the $2^{-\Delta \Delta \mathrm{CT}}$ method [72] and the β-actin gene (*ACTB*) as a reference gene.

### 2.3. Analysis of Blood Biochemical/Immunological Variables

Laboratory testing of blood samples was carried out at the St. Petersburg State Academy of Veterinary Medicine to assess the contents of 25 various biochemical/immunological parameters (see their full listing in SI Tables S2 and S3) at 1 and 7 dpi.

Protocols (as compiled in [73]) were followed to determine the serum lysozyme activity (SLA) using a photoelectric colorimetric method (including the changed temperature regime for reaction of the chicken blood serum with *Micrococcus lisodecticus*), serum bactericidal activity (SBA), and serum β-lysine activity (BLA) using a photoelectric colorimetric method. The discrete sedimentation method [74] was exploited to detect immunoglobulin levels of the classes IgA, IgM, IgG1, and IgG2.

### 2.4. Next-Generation Sequencing (NGS) of Bacterial Community Profiles

To explore metagenomic profiles of the bacterial community in the cecum of laying hens at 1 and 7 dpi, NGS technology was employed using total bacterial DNA. The latter was extracted from the cecal content samples using the Genomic DNA Purification Kit (Thermo Fisher Scientific Inc., Waltham, MA, USA) according to the manufacturer’s instructions.

Amplification for the subsequent NGS was performed using a Verity thermal cycler (Life Technologies, Inc., Carlsbad, CA, USA) and the following eubacterial PCR primers (Integrated DNA Technologies, Inc., Coralville, IA, USA): 343F (5′-CTCCTACGGRRSGCAGCAGAG-3′) and 806R (5′-GGACTACNVGGGTWTCTAAT-3′) flanking the V1V3 region of the 16S rRNA gene. Metagenomic sequencing was performed using a MiSeq genomic sequencer (Illumina, Inc., San Diego, CA, USA) and MiSeq Reagent Kit v3 (Illumina, Inc.). Read libraries with a total of 57,229 and 82,645 sequences were produced using samples collected at 1 and 7 dpi, respectively. Minimum, average, and maximum read lengths for 1- and 7-dpi samples were 188, 246.37 ± 3.58, 251 and 188, 239.60 ± 17.75, and 251 base pairs, respectively. Chimeric sequences were excluded from the analysis using the USEARCH 7.0 program [75]. Processing of the obtained reads was carried out using the CLC Bio GW 7.0 bioinformatics platform (Qiagen, Venlo, The Netherlands) and included overlapping, quality filtering (QV > 15), and primer trimming. Taxonomic affiliation of microorganisms up to the genus level was determined using the RDP Classifier program [76].

### 2.5. Mathematical and Statistical Analyses

Variance analysis (as described in [77]) was employed for the mathematical and statistical analysis of the data using Excel XP/2010-based computation, and mean values were compared using Tukey’s honestly significant difference (HSD) test and the TukeyHSD function in the R Stats Package [78].

Using the studied biochemical/immunological indices, cluster analysis of the four subgroups was performed by building a matrix based on squared Euclidean distances between objects at the two time points. For matrix aggregation, the Ward’s hierarchical agglomerative clustering was applied [79], and bootstrapping validation was performed using 1000 iterations. Alternatively, the Unweighted Pair Group Method with Arithmetic mean (UPGMA) method with bootstrapping validation was used on a matrix of Euclidean distances between objects. To calculate approximately unbiased (AU) *p*-values and bootstrap probability (BP) values, the pvclust software package for R was used [80]. Clusters with AU values more than 95% were considered as significant ones. For interpretation, the optimal number of clusters was chosen using the Elbow method [81] and factoextra package for R [82].

For the biodiversity analysis of cecal bacterial communities in laying hens within samples, the parameters of their alpha diversity were determined, which characterizes the abundance of species within communities [83]. In particular, microbiota diversity in the chicken cecum in the four subgroups was evaluated using the direct count of taxa, i.e., operational taxonomic units (OTUs), as well as Shannon index in the Past software program [84,85]. The Shannon (entropy) index (*H*), meant to quantify the accumulation of entropy (uncertainty or content of information) in a complex community and ranges between 0 for one-taxon communities to higher values for multiple-taxa communities (each taxon being with few and more individuals), was calculated by the following formula (as provided in [86]):

$$H=-\underset{i}{\sum }\frac{n_{i}}{n}\ln\frac{n_{i}}{n}$$

where *H* is the diversity (in bits), and *n_i_*/*n* is the proportional abundance of the species computed as number of individuals of a species (*n_i_*) divided by the total number of individuals found (*n*).

Due to the fact that the values of biological diversity parameters vary according to the number of sequenced reads in each sample, reflecting the depth of sequencing, we evaluated the diversity using normalized data for the smallest number of sequences present in a sample.

In addition, beta diversity, i.e., diversity between samples/communities/habitats, or the degree of change in species composition by gradients [83], was evaluated. For this purpose, we examined the microbial diversity of the samples using the principal components analysis (PCA) as implemented in the EMPeror tool [87], Weighted UniFrac metric [88], and the QIIME software package [89]. Using the cecal microbiome profiles at the two time points, we also exploited the Ward’s hierarchical agglomerative clustering [79].

Furthermore, diversity of the chicken cecal metagenomic communities at the level of orders and higher taxonomic ranks was also evaluated graphically in the form of a heat map. Construction of the microbiome profile heat map was based on a phylogenetic matrix of grouped samples using the Plotly package for R [90]. Biodiversity of the cecal microbiome of laying hens at lower taxonomic levels was assessed using the MEGAN program, version 6.15.2 [91], and log-normalized data were used to plot the graphs.

To estimate relationships between treatments and studied characters in the subgroups, regression models using the basic functions in R [92], as well as the Pearson’s correlation coefficient and three-way ANOVA tests [92], were employed.

## 3. Results

### 3.1. Changes in Gene Expression

The DGE analysis showed that, at 1 dpi, a downregulation of the *AvBD10* gene (up to 1.92 times; *p* ≤ 0.01) was observed in all experimental subgroups relative to the negative control S-I (SI Figure S1a). At this time point, the administration of phytobiotic Intebio^®^ in S-III also contributed to a downregulation of the *AvBD10* gene as compared with S-I (*p* ≤ 0.01), but, in S-IV, it led to a higher expression of this gene than that in S-II (*p* < 0.05). At 7 dpi, an upregulation of this gene was found in all experimental subgroups in comparison with S-I (*p* < 0.01; SI Figure S1b).

At 1 dpi, the *IL6* gene was downregulated in S-II and S-III and upregulated in S-IV (*p* < 0.05; SI Figure S1a). At 7 dpi, a downregulation of the *IL6* gene was observed in S-II, and there its expression was elevated in S-III and S-IV as compared with the two other subgroups (*p* < 0.05; SI Figure S1b). Remarkably, S-II showed a downregulation of *IL6* by 2.2 times at 1 dpi (*p* < 0.05) and by 2.7 times at 7 dpi (*p* < 0.05) relative to S-I. The *IL8L1* gene was significantly upregulated in all the experimental subgroups—up to 15.3 times in S-IV in comparison with S-I (*p* < 0.001; SI Figure S1a). At 7 dpi, the *IL8L1* gene upregulation in the experimental subgroups as compared with S-I was not as pronounced as at 1 dpi and did not exceed 1.82 times (*p* < 0.01; SI Figure S1b).

A significant downregulation of the *SLC5A1* gene was shown at 1 dpi in S-II and S-IV relative to S-I (by 2.8 and 2.9 times, respectively; *p* ≤ 0.05; SI Figure S1a). At 7 dpi, the expression level of this gene was restored, and there was even a significant upregulation in S-II and S-IV (*p* ≤ 0.05; SI Figure S1b). The *CA2* gene expression was significantly higher at 1 dpi in all the experimental subgroups as compared with S-I (*p* < 0.05; SI Figure S1a). At 7 dpi, this gene was also characterized by upregulation, with its sharp activation being detected in S-II and S-III by 388.0 and 488.9 times, respectively, relative to S-I (*p* < 0.001; SI Figure S1b). A significant decline in the *CALB1* gene expression was found in S-IV (*p* < 0.05; SI Figure S1a), while, at 7 dpi, there was a significant upregulation of this gene by 1.7 times in S-II (*p* ≤ 0.05) and by 3.4 times in S-III in comparison with S-I (*p* < 0.01; SI Figure S1b).

The *RARRES1* gene expression remained unchanged in all the experimental subgroups at 1 dpi (SI Figure S1a). However, there was a significant upregulation of this gene at 7 dpi in response to the *Salmonella* challenge in S-II as compared with S-I (*p* ≤ 0.05; SI Figure S1b). At 1 dpi, the *BPIFB3* gene regulation was significantly reduced in S-III (*p* < 0.05) and increased in S-IV (*p* < 0.05; SI Figure S1a). By 7 dpi, a significant upregulation of this gene was found in S-III and S-IV, reaching a factor of ~3100 in the latter (*p* < 0.05; SI Figure S1b).

**Figure 1:**
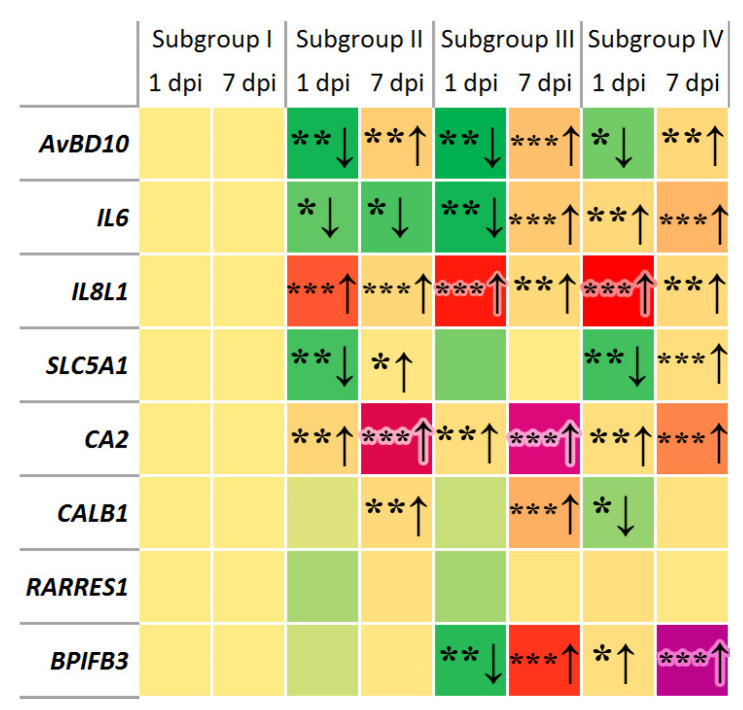
Heat map showing the expression of the chicken genes involved in response due to the *Salmonella enterica* serovar Enteritidis (SE) challenge and/or phytobiotic intake. Red and green squares correspond to significant gene up- (↑) or downregulations (↓) relative to the negative control (S-I) at *p* < 0.05 (*), *p* < 0.01 (**), and *p* < 0.001 (***). Yellow squares and other squares with no “*” mean, respectively, the basal expression level in S-I (negative control) or no significant changes in the experiment subgroups. dpi: days post-inoculation.

The heat map in Figure 1 represents the generalized pattern of DGE observed during the entire study in the negative control and experimental subgroups, depending on the *Salmonella* challenge and/or Intebio^®^ addition. Overall, a significant downregulation was detected at 1 dpi and upregulation at 7 dpi for the *AvBD10* gene in all subgroups, for *IL6* in S-III, for *SLC5A1* in S-II and S-IV, and for *BPIFB3* in S-III relative to S-I. At both 1 and 7 dpi, the *IL6* gene showed a significant downregulation in S-II, whereas there was a significant upregulation of *IL6* in S-IV, *IL8L1* and *CA2* in S-II to S-IV, and *BPIFB3* in S-IV. The expression of *CALB1* in S-II and S-III and that of *RARRES1* in S-II did not differ significantly from that in S-I at 1 dpi and changed to an increased regulation at 7 dpi. In addition, the *CALB1* gene expression decreased at 1 dpi in S-IV was restored at the level of S-I at 7 dpi (Figure 1).

### 3.2. Dynamics of Blood Biochemical/Immunological Variables

By studying alterations in the 25 blood biochemical/immunological parameters in layers, it was found that, at 1 dpi, there was a lower albumin (ALB) content in the challenged subgroups S-II and S-IV than that in the nonchallenged subgroups S-I and S-III (*p* < 0.01; SI Table S2). At 7 dpi, S-II was still characterized by the lowest ALB content (*p* ≤ 0.05; SI Table S3).

The urea (UR) concentration was the lowest in S-II and the highest in S-IV at 1 dpi in comparison with S-I, while, by 7 dpi, only S-II was different from S-I and superior to all other subgroups, with almost similar patterns for the inter-subgroup differences being identified for blood urea nitrogen (BUN) (*p* ≤ 0.05; SI Tables S2 and S3). The creatinine (CR) content in the blood of all the studied subgroups was, at both time points, below the normal reference range (SI Tables S2 and S3). However, S-III and S-IV showed, at 1 dpi, an increased CR content by 1.2 and 1.3 times, respectively, as compared with S-II (*p* < 0.05), while this parameter was superior in S-II at 7 dpi relative to all other subgroups. In addition, there was a lower ALT activity at 1 dpi in S-II and S-III, whereas, at 7 dpi, a significantly greater ALT level was observed in S-II and S-IV than that in S-I (*p* ≤ 0.05). In S-IV, this parameter exceeded the upper limit of the reference range for healthy birds and was 2.7 times greater than that in S-III (*p* ≤ 0.001).

At 1 and 7 dpi, there was a lower content of aspartate aminotransferase (AST) in S-III and S-IV fed Intebio^®^ than that in S-I and S-II fed a normal diet (*p* < 0.05). On the other hand, there was the greatest AST activity at 7 dpi in S-II relative to all other subgroups and in S-IV as compared with S-III (*p* ≤ 0.05). A greater activity of alkaline phosphatase (ALP) was demonstrated at 1 dpi in S-III and S-IV relative to S-I and S-II, while it lowered by 7 dpi in S-II and S-III in comparison with S-I (*p* ≤ 0.05) and was deranged, being below the reference range at both time points (SI Tables S2 and S3). It was also found that, at 1 dpi, there was an elevation of the alpha amylase (AMY) content in S-II and S-IV as compared with S-I and S-III, whereas its activity by 7 dpi was lower in S-II than that in S-I (*p* ≤ 0.05). The GLUC concentration at 7 dpi in S-II to S-IV was also lower than that in S-I (*p* ≤ 0.05) and was, on the whole, below the reference range (SI Tables S2 and S3).

The total cholesterol (CHOL) concentration at 1 dpi was the lowest in S-II and S-IV and the highest in S-III, while it did not differ at 7 dpi in S-II and S-III from that in S-I and was superior in S-IV (*p* ≤ 0.05). The calcium total (CaT) level at 1 dpi was inferior in S-II and S-IV to that in S-I and S-III (*p* ≤ 0.05). The phosphorus (PHOS) content was deranged and lower at 1 dpi in the blood of challenged birds in S-II and S-IV by 1.4 and 1.6 times, respectively, as compared with S-I and S-III (*p* < 0.05), whereas it decreased in S-II and S-III (*p* ≤ 0.05) and grew in S-IV up to the level of S-I at 7 dpi. There was a greater bilirubin (BIL) concentration at 1 dpi in S-III and S-IV and a lower one in S-II than that in S-I. At 7 dpi, it was still the greatest in S-IV but declined in S-III below the BIL level in S-I (*p* ≤ 0.05).

A significant drop of the uric acid (URA) content in the blood was observed at 1 and 7 dpi in the challenged subgroups S-II and S-IV relative to the nonchallenged subgroups S-I and S-III (*p* < 0.01). The SLA in S-II and S-III was greater at 1 dpi than that in S-I, and it was elevated in all the experimental subgroups relative to S-I by 7 dpi (*p* ≤ 0.05). There was an increased SBA level in S-IV by a factor of 1.3 at 1 dpi and by a factor of 1.7 at 7 dpi in comparison with S-II (*p* < 0.05), whereas the intermediate levels of SBA were found out in S-I and S-III. The BLA was lower at 1 dpi in S-II and S-III than that in S-I and S-IV and changed at 7 dpi so that it was the lowest in S-II and the highest in S-IV as compared with S-I (*p* ≤ 0.05).

The immunoglobulin activity also varied between the four subgroups and two time points. In particular, a declined IgA level in S-II and elevated one in S-III and S-IV were identified at 1 dpi, while it lowered in S-III and S-IV by 7 dpi to below the IgA activity in S-I (*p* ≤ 0.05). The challenged S-II and S-IV were characterized by the lowest IgM activity at 1 dpi, with its greater levels being found at 7 dpi in S-II to S-IV relative to S-I (*p* ≤ 0.05). Additionally, a greater IgM level was determined at 7 dpi in S-IV relative to S-III (*p* < 0.01). As for the IgG1 levels at 1 dpi, S-II and S-IV were also inferior to S-I (*p* ≤ 0.05), and S-III was superior to the three other subgroups, while the declined IgG1 activity was observed at 7 dpi in S-II and S-III in comparison with S-I and S-IV (*p* ≤ 0.05). The IgG2 activity was lower in S-II and S-IV at 1 dpi and greater in S-II at 7 dpi as compared with S-I (*p* ≤ 0.05). No significant differences were found between the subgroups and time points in the contents of the total protein and globulins.

Using a whole set of the studied biochemical/immunological parameters and plotting the subgroup clustering, distinctive differences between the four subgroups were demonstrated (SI Figure S2). As confirmed by two tree-building methods, the Ward’s hierarchical agglomerative clustering, and UPGMA, S-III and S-IV were, at 1 dpi, the most similar to each other, and S-II was the most distinguishing (SI Figure S2a,e), while the tree topology of the four subgroups essentially changed by 7 dpi, so that the two challenged and two nonchallenged subgroups grouped into two separate clusters (SI Figure S2b,f). Normalization of the datasets led to a different Ward’s clustering pattern (SI Figure S2c,d), showing the formation of the challenged and nonchallenged subgroup clusters already at 1 dpi.

An additional Pearson’s correlation coefficient test resulted in demonstrating a significant positive correlation between the *IL6* gene expression and SBA at 1 (*r* = 0.88; *p* < 0.01) and 7 dpi (*r* = 0.76; *p* < 0.01), *AvBD10* expression and BLA at 1 dpi (*r* = 0.9; *p* < 0.001), *IL6* expression and BLA at 7 dpi (*r* = 0.81; *p* < 0.01), and *IL8L1* expression and IgA activity at 1 dpi (*r* = 0.71; *p* < 0.01). At 7 dpi, the expression of *IL6* (*r* = 0.63; *p* < 0.05) and *IL8L1* (*r* = 0.73; *p* < 0.01) positively correlated with the IgM activity in the blood.

### 3.3. Cecal Microbiome Diversity Characterization

To assess the alpha diversity of the cecal bacterial communities within the subgroups studied, the mean values of the appropriate parameters, OTUs, and Shannon index were calculated. As shown in Table 1, when the birds were challenged with SE and/or fed the diet supplemented with Intebio^®^, there was no significant change in the number of OTUs within one time point in any subgroup. There was a small but significant recession in the Shannon index estimate at 1 dpi in S-IV as compared with S-I (*p* < 0.01). At 7 dpi, there were no differences between the subgroups for both alpha diversity parameters; however, the number of OTUs significantly lowered in all subgroups relative to 1 dpi (*p* < 0.05).

To characterize the beta diversity differences between the four subgroups, a PCA was performed (SI Figure S3). As can be seen from SI Figure S3a, the PC1 component allowed to explain 48.57% of the variance in the 1 dpi dataset, while, respectively, 19.96% and 9.46% of the variance were explained by PC2 and PC3, meaning that the first three principal components explained a total of 77.99% of the data information at describing the shifts that occurred in the microbiome profiles of the four subgroups at 1 dpi.

The PCA-based comparison of the cecal microbiota diversity in the four subgroups (SI Figure S3a) showed that, judging from the microbiome profiles, layers from S-IV and, to some extent, S-II formed separate clusters. The greatest displacement along the PC1 axis was observed in the samples from S-IV, while the other three subgroups were drawn to the PC2 axis. Clustering in S-II and S-III was less pronounced than that in S-I and S-IV. Apparently, the composition of chicken microbiomes in S-III also had more affinity with that in S-I. The beta diversity of the cecal microbiota at 7 dpi can be described as shown in SI Figure S3b. The cluster formation in S-II and S-III was less pronounced as compared with S-I and S-IV. Subgroups IV, I, III, and II were consistently lined up along the PC1 axis with the smallest clustering effect along two other axes. The clusters of S-I and S-III were close to each other and occupied an intermediate position between the other two subgroups.

**Figure 2:**
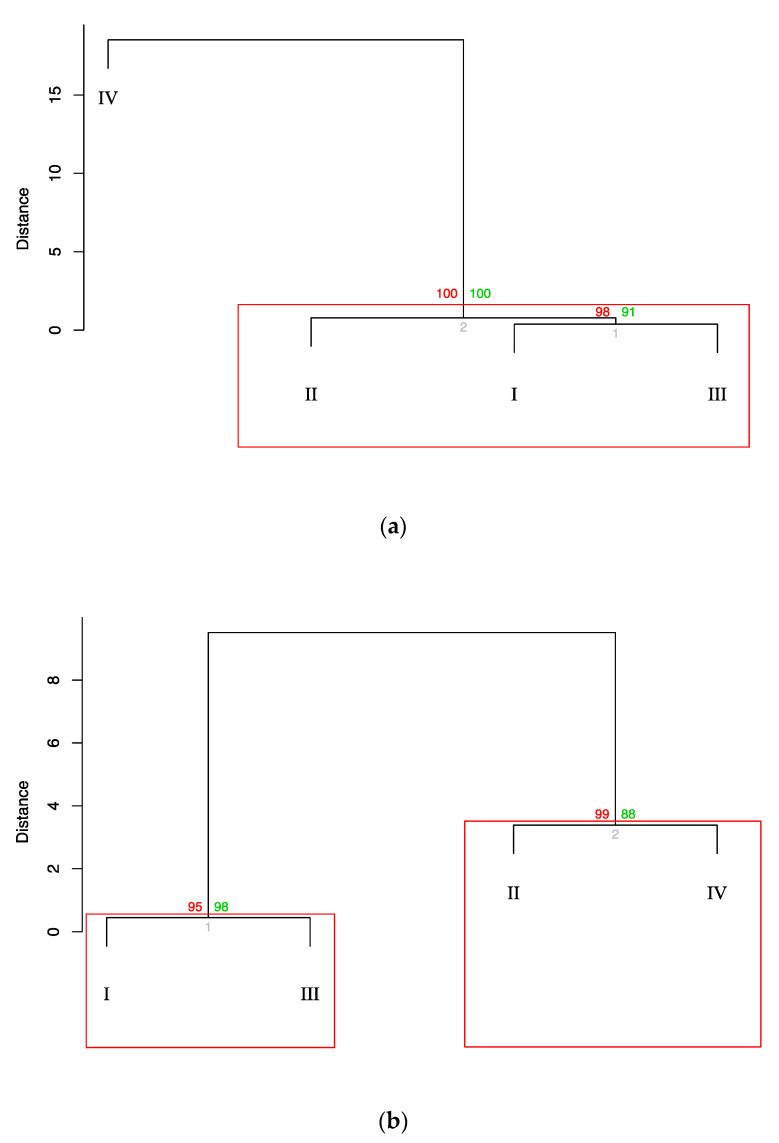
Ward’s hierarchical agglomerative clustering based on the chicken cecal microbiome profiles at 1 (**a**) and 7 dpi (**b**) and using a matrix of squared Euclidean distances between objects. Bootstrapping validation was performed using AU (approximately unbiased) *p*-values (%) and BP (bootstrap probability) values (%) shown with red and green estimates, respectively. Clusters with AU *p*-values greater than 95% are placed within red rectangles. Subgroups: I (negative control), II (SE challenge), III (Intebio^®^ intake), and IV (Intebio^®^ intake + SE challenge).

To achieve a better resolution of the inter-subgroup relationships, we proceeded with the Ward’s hierarchical agglomerative clustering-based analysis of the chicken cecal microbiomes at 1 and 7 dpi using squared Euclidean distances (Figure 2) that demonstrated a clear separation of the subgroups into specific dendrogram clusters with the appropriate significant AU/BP values, although the inter-subgroup distances within the clusters were mainly quite small. In particular, at 1 dpi, the microbiomes of S-I and S-III merged into a common cluster (at a ~0.5 distance; Figure 2a). S-IV was remotely allocated on a separate branch at a considerable distance from the other subgroups. S-II also formed a separate branch, but it clustered with S-II and S-III, being located at a much smaller distance (~1) to them than S-IV. At 7 dpi (Figure 2b), the microbiomes of S-I and S-III were closest to each other, combining in one cluster (at ~0.05), and then, S-II and S-IV grouped into the second cluster (at ~3.5), while these two clusters were located much farther relative to each other.

Further, we performed Ward’s clustering by placing together the cecal microbiomes at 1 and 7 dpi (SI Figure S4) that generally confirmed the pattern of dendrograms built separately for the two time points (Figure 2), the within-cluster distances between subgroups being mainly smaller at one time point.

**Figure 3:**
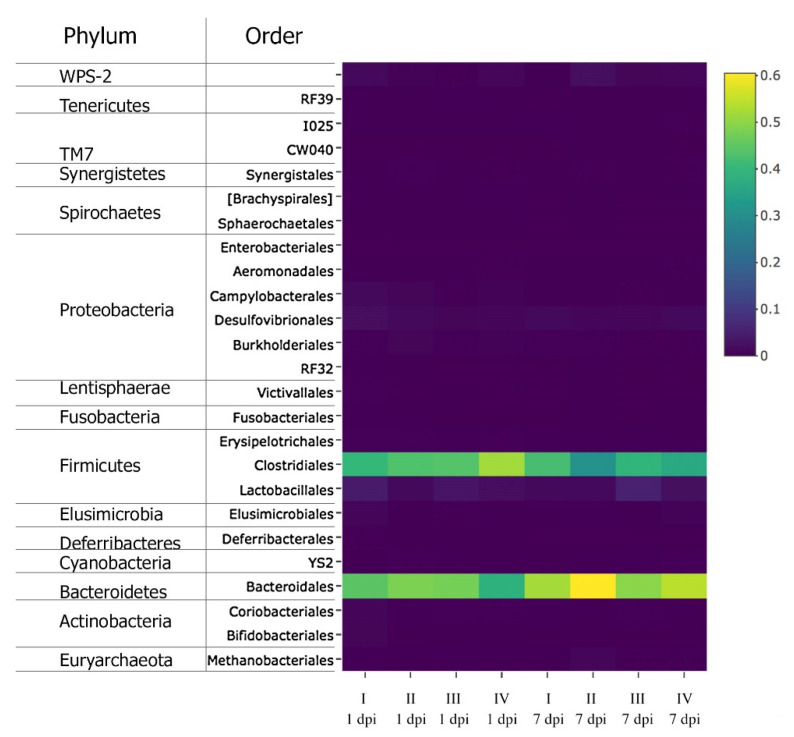
Heat map of the cecal microbiome biodiversity in laying hens at 1 and 7 dpi at the level of orders and higher taxonomic ranks in the subgroups: I (negative control), II (SE challenge), III (Intebio^®^ intake), and IV (Intebio^®^ intake + SE challenge). The columns correspond to subgroups and the rows to bacterial taxa. On the color scale, a value of 0.1 is equal to 10% of the representation of microorganisms in the community.

In Figure 3, diversity profiling of the cecal microbiota at the level of orders and higher taxonomic ranks is presented in the form of a heat map. As seen from Figure 3, 13 phyla and 24 orders of attributable microorganisms were identified in the cecal metagenomic communities of the chickens.

At 1 dpi, microflora at the level of phyla was dominated by representatives of Firmicutes and Bacteroidetes (Figure 3). Among the phylum Firmicutes, a significantly greater proportion was determined for the order Clostridiales bacteria, with their contents ranging between the minimum of 40.3% ± 3.61% and the maximum of 52.0% ± 4.39% (at *p* < 0.05 for a pairwise comparison between the subgroups). In chickens challenged with *Salmonella* (S-II) and fed Intebio^®^ (S-III), there were no significant changes in the number of these microorganisms. A distinct elevation in the bacterial content of the order Clostridiales was observed in S-IV (52.0 ± 4.39%) as compared with S-I (*p* < 0.05). Among the prevailing microorganisms of the phylum Bacteroidetes, there were the order Bacteroidales representatives (38.3% ± 3.85% to 48.3% ± 3.25%; *p* < 0.05). A greater number of these microorganisms was found in S-II and S-III than that in S-I (*p* < 0.05). In contrast, there was a decline in the contents of these bacteria in S-IV.

Bacteria from the phyla Firmicutes and Bacteroidetes were also abundant at 7 dpi (Figure 3). The greatest percentage of the bacteria from the order Clostridiales was found in S-I (42.0 ± 1.33%), their proportion being smaller in S-II (30.8% ± 0.53%; with *p* < 0.05 for the differences between the subgroups). There was also a slight increase in the number of bacteria from the order Lactobacillales in S-III (5.5% ± 4.95%) as compared with the minimum value in S-I (1.5% ± 0.26%), and an augmentation in Bacteroidales in S-II (60.5% ± 0.59%) relative to S-III (49.7% ± 6.59%), but these differences between the subgroups were insignificant. As evident in SI Figure S5a, among the order Bacteroidales, the most pronounced shifts in the number of bacteria at 1 dpi in the subgroups were observed for the dominant species of *Bacteroides barnesiae* (*p* < 0.01).

The number of the genus *Bifidobacterium* representatives—among which, *B. longum* dominated—was reduced in challenged chickens: by 10.5 times in S-II and by 12.5 times in S-IV relative to S-I (*p* < 0.01; SI Figure S5a). There was a similar scaling down in the contents of the order Lactobacillales, represented predominantly by the genus *Lactobacillus* microorganisms, in S-IV relative to S-I (*p* < 0.05). The content of the genus *Methanobrevibacter* archaea decreased in the cecum of layers by 1.2 times in S-II (*p* ≤ 0.05) and by 1.9 times in S-IV (*p* < 0.05) as compared with S-I (SI Figure S5a). A growth in the content of the genus *Campylobacter* bacteria occurred by 5.5 times in S-II (*p* < 0.05) and by 5.6 times in S-IV (*p* ≤ 0.01) relative to S-I. In addition, we detected in the chicken cecal microbiota some very small number of candidate division TM7 bacteria (up to 0.29% ± 0.02%; *p* < 0.05) and candidate division WPS-2 representatives (up to 1.4% ± 0.06%; *p* ≤ 0.05).

At 7 dpi (SI Figure S5b), the disappearance of the *B. longum* bacteria in S-II, S-III, and S-IV was observed. In S-I, their number also dropped by about 100 times relative to 1 dpi (*p* < 0.05). Differences between the subgroups in the content of the genus *Lactobacillus* microorganisms were insignificant. The genus *Bacteroides* was mainly represented by the species *B. barnesiae* and *B. plebeius*. There was a significant decline by 2.7 times in the number of *B. plebeius* in S-III as compared with S-II (*p* < 0.05). The order Clostridiales was represented by the genera *Coprococcus*, *Dorea*, *Oscillospira*, and *Ruminococcus* that demonstrated the greatest variability in the subgroups. No significant differences between the subgroups in the genera *Coprococcus*, *Ruminococcus*, and *Oscillospira* were found. There were 3.7 times more representatives of the genus *Dorea* in S-III in comparison with S-II, which had their smallest number (*p* < 0.05).

### 3.4. Effects of SE and Intebio^®^ on Egg Productivity

An analysis of the egg production traits over a 27-day egg-laying period revealed significant differences between their mean values in the four subgroups of laying hens (Table 2). In particular, S-I was significantly inferior in egg weight (64.94 g) to S-III (66.10 g; *p* < 0.05) and S-IV (66.33 g; *p* < 0.01) and superior in egg mass to S-II (547.54 vs. 479.19 g; *p* < 0.05). S-II had lower mean values than S-IV by egg weight (64.29 vs. 66.33 g; *p* < 0.01) and was relative to S-III by the number of laid eggs (7.48 vs. 8.56; *p* < 0.05) and egg mass (479.19 vs. 564.81 g; *p* < 0.01). S-III exceeded S-IV in the number of eggs (8.56 vs. 7.52; *p* < 0.05) and in egg mass (564.81 vs. 498.86 g; *p* < 0.05).

Additionally, a post-peak egg production recession was analyzed using daily egg mass values in the subgroups. Based on the decreased rate, it was found that there was a rather slower diminution of egg productivity in laying hens fed Intebio^®^, especially in S-III, in comparison with S-I and S-II (see, for details, SI File S2). A comparison of the dependences in S-I and S-II (Figure SI2-1b) suggested a stable decline in their egg mass values throughout the entire 27-day period. The comparison of S-I and S-III (Figure SI2-1c) showed that, starting from about the middle of the observation period, S-III was superior to S-I. When comparing S-I and S-IV (Figure SI2-1d), it was noted that S-IV gradually aligned with S-I (control), while S-III had a better egg production recession rate than that in S-IV (Figure SI2-1e).

### 3.5. Mathematical Analyses Combining All Studied Characters

**Figure 4:**
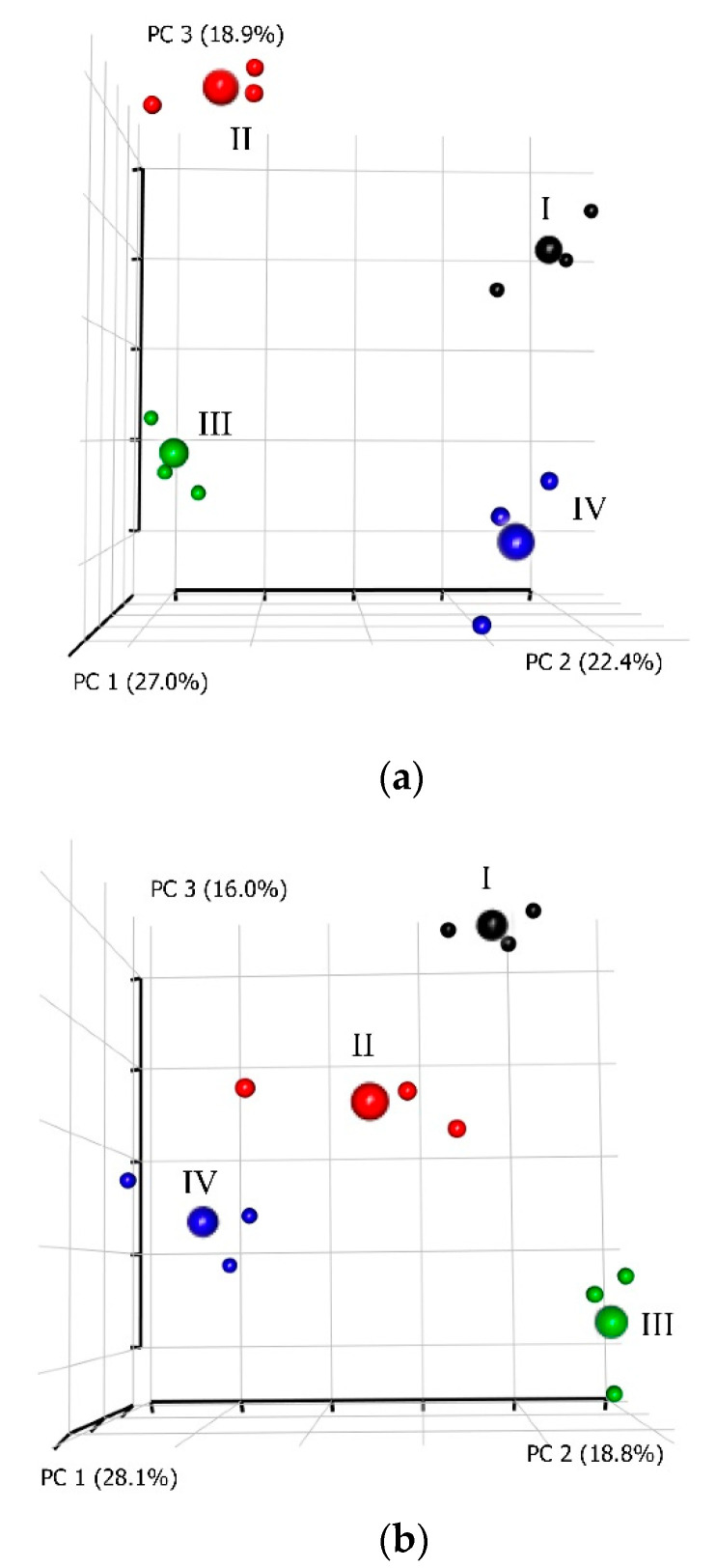
Principal components analysis of the samples by a complex of characters represented as three-dimensional graphs (one point corresponds to one hen, the mean values for each subgroup being shown as the fourth point of the larger diameter) at 1 (**a**) and 7 dpi (**b**) in the subgroups: I (negative control), II (SE challenge), III (Intebio^®^ intake), and IV (Intebio^®^ intake + SE challenge).

Ultimately, a resumptive analysis was conducted using the PCA method at the two time points, 1 and 7 dpi, taking into account all groups of characters measured in the four subgroups of laying hens (SI Figure S6 and Figure 4), including DGE; the blood biochemical/immunological parameters; cecal microbiome profiles; and three egg production traits (mean egg weight, number of laid eggs, and egg mass). Visualization using the PCA enabled us to describe the multivariate datasets, show the interrelations of the characters studied, and identify the key factors in this experiment at the two time points. To describe the complex of these characters, the first three principal components (PC) were considered.

In two-dimensional graphs, the first principal component (PC1) at 1 dpi (SI Figure S6a) was most affected by such blood biochemical/immunological factors as concentrations of ALB, PHOS, and URA, and the immunoglobulin activity (IgG1, IgG2, and IgM), as well as the egg productivity traits, i.e., mean egg weight and, to a lesser extent, number of laid eggs and egg mass. The serum BLA negatively correlated with SLA had the greatest impact on PC2. PC3 was affected by IgA, BIL, and CR (SI Figure S6b).

The nonchallenged subgroups S-I and S-III and the challenged subgroups S-II and S-IV occupied mutually opposite positions along the PC1 axis (SI Figure S6a). Probably, PC1 was associated with the factors responsible for the development of an infection process at 1 dpi, defining the lowered content of serums ALB, PHOS, and URA and immunoglobulins IgM, IgG1, and IgG2, as well as a decline in the egg production traits. Using PC2, one can observe that S-I and S-III were spread along the PC2 axis. The SLA in S-III was higher than that in S-I. PC3 was likely to reflect the interactions of two factors, SE challenge, and administration of phytobiotic Intebio^®^. The PC3 factors did not have a significant impact on S-I and S-III. S-IV differed from S-II by a greater content of IgA, BIL, and CR.

At 7 dpi, there was a change in the limiting factors (SI Figure S6c,d), with PC1 being mainly affected by UR, BUN, CR, and SBA. The mutual arrangement of S-II and S-IV relative to the PC3 axis, as compared with 1 dpi, altered to the opposite one. S-II was distinguished by a high content of UR, BUN, and serum CR. Probably, PC1 reflected a reserve capacity of the hen’s organism. The greatest contribution to PC2 at 7 dpi was made by the contents of ALT, BIL, and IgM. S-IV differed from S-I and S-III by greater values of these characters. The negatively correlated with each other ALP (that was in S-III lower than in S-I) and SLA contributed the most to PC3, while PC3 had a lesser effect on S-II and S-IV.

By the complex of characters in layers of the four subgroups, their comparisons based on PCA clustering in two- (SI Figure S6) and three-dimensional graphs (Figure 4) showed that conjugate changes in the studied characters at 1 dpi in chickens from S-I and S-II led to their separation into single remote clusters. There was a rather detached and, at the same time, close location of S-III and S-IV that lay on the same plane formed by the axes PC1 and PC2, suggesting that, by the complex of characteristics, S-III had a significant affinity with S-IV relative to the main factors of PC3, including the IgA, BIL, and CR contents (Figure 4a).

At 7 dpi, clustering on the basis of the studied character sets also had fairly distinct outlines for all four subgroups (Figure 4b). Hereby, S-III showed, on a three-dimensional graph, the maximum distance from other subgroups, while S-II and S-IV were located in proximity to each other. S-I was also located at a somewhat remote distance from the other subgroups.

Since one of the established factors that influenced PC1 at 1 and 7 dpi was the IgM activity, we employed a regression model to determine the degree of the effect of Intebio^®^ intake on the IgM level. The IgM activity was closely related to a number of its explaining predictors as follows: content of ALP, AST, and CHOL, along with the factors of SE infection and Intebio^®^ supplementation, each of which contributed to the shift in the level of this immunoglobulin. The following optimal and reliable multicharacter model was developed, with all statistical coefficients being significant (*p* = 0.007 for intercept, *p* < 0.001 for predictors, adjusted *R*^2^ = 0.9339 for this model, *F* statistic = 66.03, and *p* = 6.06 × 10^−11^ for the general significance level of the model):

(1) X=−1.671+0.624A+0.350B+ 0.007C−0.007D + 1.146E

where *X* is the IgM level, *A* is the Intebio^®^ intake factor, *B* is the SE infection factor, *C* is the AST level, *D* is the ALP level, and *E* is the CHOL content.

In the above model, the interaction of factors was not taken into account, and each predictor contributed a certain share due to the appropriate coefficient value. Considering Formula (1), the IgM activity depended on a constant equation member meaning a shift equal to −1.671. The diet factor coefficient (0.624) was greater than that for the SE infection factor (0.350), reflecting a greater increase of the IgM value in the subgroups fed the Intebio^®^ supplement vs. normal diet than that in the subgroups infected with SE vs. nonchallenged hens. Additionally, growth in the IgM content was slightly positively associated with AST (0.007) and, especially, with CHOL (1.146), whereas it had a somewhat negative relationship with ALP (−0.007), meaning a slight decrease in the ALP level while the IgM activity was elevated. AST and ALP had the lowest coefficients, suggesting their smaller contribution to the IgM level. The largest factor was the CHOL content (1.146), and its effect on the IgM level alteration could be expressed more decidedly in contrast to a more discreet transition due to the SE infection and Intebio^®^ intake factors.

Based on the above multicharacter model (1) for IgM predictors, and using the Pearson’s correlation coefficient test, we determined a significantly close correlation between CHOL and IgM (*r* = 0.70; *p* < 0.01); however, no significant correlation was established between the other predictors and IgM.

As a result of the three-way ANOVA tests, we found that the factors of SE challenge and phytobiotic intake had a significant effect on the levels of IgM, CHOL, AST, and ALP (*p* < 0.05). The factor of the two time points in relation to the blood indices measured had a significant effect on the IgM, CHOL, and AST (*p* < 0.05) but did not affect the ALP activity (*p* > 0.05).

## 4. Discussion

### 4.1. Gene Expression Changes in Response to SE and Phytobiotic

As was shown in previous studies (e.g., in broilers [93]), the most pronounced alteration in the expression level of genes associated with the immune system was found 1 day after the challenge with *Salmonella*. In the present study, we also investigated the immune response of laying hens to SE via DGE at 1 dpi, as well as at the 7-dpi time point, in order to observe the long-term challenge effects. For this purpose, we selected some key genes related to immunity (*AvBD10*, *IL6*, *IL8L1*, and *BPIFB3*); the transport of nutrients and ions in the intestine; and metabolism (*SLC5A1*, *CA2*, *CALB1*, and *RARRES1*). Moreover, it was desirable in terms of tissue-specific expression and for comparative purposes to juxtapose these chicken genes with their homologs in mammals (including humans), since most of these genes were shown to be expressed, for example, in the human GIT [51], which we also discuss below where appropriate.

As a result of our studies, it was shown that the expression of the *AvBD10* gene associated with the synthesis of gallinacin-10 and the anti-inflammatory immune response [94] was downregulated at 1 dpi in all experimental subgroups relative to S-I (up to 1.92 times; Figure 1 and SI Figure S1a). Previously, a reduction of the *AvBD10* expression in the cecum by 2.04 times was observed in an experiment on the infection of chickens with *S. enterica* serovar Pullorum as compared with the nonchallenged subgroup [95]. Similarly, when the chickens were infected with *Salmonella*, a lower defensin gene expression was detected in susceptible birds sensitive to *Salmonella* infection in comparison with resistant ones, and differences in the level of immunity-related DGE between the susceptible and resistant chicken lines were demonstrated [96]. In our experiment, an upregulation of the *AvBD10* gene was found at 7 dpi in all the experimental subgroups relative to S-I (Figure 1 and SI Figure S1b). In all likelihood, this gene is involved in providing a resistance to SE in the studied laying hens, but its activation occurred later than 1 dpi. Interestingly, S-III and S-IV fed Intebio^®^ also demonstrated, at 1 dpi, a significant *AvBD10* downregulation in comparison with S-I. Despite this, the level of this gene expression in S-IV was significantly higher than that in S-II, suggesting some positive effect of Intebio^®^ on the *AvBD10* expression in challenged hens. This seemed to be a positive phenomenon relevant to the bacteriostatic [43] and anti-inflammatory response [94] exhibited by this gene. The anti-inflammatory effect of the EOs was also noted in other studies [16,17].

We also found an upregulation of the *IL6* gene at 1 and 7 dpi in S-IV relative to S-I, S-II, and S-III (Figure 1 and SI Figure S1). S-II showed a significant *IL6* downregulation at both 1 and 7 dpi relative to S-I. Apparently, this gene in our experiment was significantly suppressed in response to the SE challenge in S-II, whereas the dietary supplementation with Intebio^®^ in S-IV could have a positive effect on the elevated *IL6* expression level and, presumably, on immunity as a whole. IL6 is known to play a central role in defense mechanisms, hematopoiesis, and acute phase reactions and is part of the innate protective immune responses [97,98,99]. On the other hand, the hyperproduction of proinflammatory cytokines such as IL6 may have negative effects in animals [100,101,102]. The biological significance/role of IL6 has been widely studied in mammals [103], including humans [51]. In particular, this homologous gene is known to be highly activated in the human gall bladder, appendix, and esophagus, while being expressed in other GIT organs [51]. Therefore, we may consider this homologous gene expression as one of the important protective elements of the chicken cecum, as well as in the digestion of amniotes, in general.

As for *IL8L1*, a gene associated with chemokine synthesis involved in signaling between immune cells [35], a drastic elevation in its expression level was observed at 1 dpi in all the experimental subgroups in comparison with S-I. There was also a significant *IL8L1* upregulation in S-III and S-IV relative to S-II, suggesting that this could be because of the Intebio^®^ intake and may point out a positive role of the phytobiotic in strengthening the protective barriers of the hen’s body. The proinflammatory cytokine IL8 is a mediator of the inflammatory process [104]. Although inflammation can be indicative of an immune response that inhibits the infection process, increased levels of proinflammatory mediators can also have negative consequences, including tissue damage and decreased productivity [105]. Previously, when infecting Ross 308 broilers with SE, a significant increase in the expression level of chemokines *IL8L1* (CXCLi1) and *IL8L2* (CXCLi2) was identified in the cecal tonsils (by 3.13 times) in comparison with nonchallenged birds, as well as infection with other tested *Salmonella* serovars [106]. In addition, in another experiment [93], there was an upregulation of genes associated with the synthesis of CXC chemokines, including *IL8L1*, in the small intestine of chickens of two lines (fast-growing line A and slow-growing line B) in response to an infection with SE. At 7 dpi (Figure 1 and SI Figure S1b), we also found a significant *IL8L1* upregulation in all the experimental subgroups, although it was less pronounced than at 1 dpi and did not exceed 1.82 times as compared with S-I. Other studies [107,108] also reported a growth in the number of cytokines and immune proteins such as IL6, IL8, IL1B, IL12, IL17, IL18, IL22, IL23, IFNG, and LITAF after the infection of chickens with *Salmonella*, which, to some extent, echoes our results. The homologous human *IL8* gene is also highly expressed in the appendix (with the second-highest level among the studied organs and tissues), gall bladder, and liver [51], suggesting its important defensive role in the GIT of higher vertebrates.

We identified, at 1 dpi, a significant *SLC5A1* downregulation in the challenged subgroups relative to S-I: by 2.8 times in S-II and by 2.9 times in S-IV (Figure 1 and SI Figure S1a). SLC5A1 is a membrane protein, a sodium-dependent GLUC cotransporter, which is part of the GLUC carrier family, and is a high-affinity Na^+^/GLUC transporter and ensures almost all uptake of the carbohydrates in the small intestine [52]. The transport of GLUC and galactose through small intestinal enterocytes provides an effective ionic homeostasis and is the first step in the absorption of carbohydrates and feeds [109]. It is likely that intestinal infection caused by *Salmonella* and resulting from the interaction between various virulence factors of this pathogen and host defense mechanisms can alter the transport of nutrients in the GIT, aggravating inflammation and causing diarrhea and malabsorption [29,30]. The downregulated *SLC5A1* expression we observed in challenged hens may be associated with the above negative intestinal effects related to *Salmonella* infection. By 7 dpi, however, the *SLC5A1* expression level was restored and even upregulated in both challenged subgroups: S-II and S-IV (Figure 1 and SI Figure S1b). In humans, high levels of this homologous gene expression were detected under normal conditions—most of all, in the duodenum, small intestine, and gall bladder—whereas it was also expressed to a lesser degree in the colon and appendix [51].

Our study demonstrated a small but significant *CA2* gene upregulation at 1 dpi in all the experimental subgroups as compared with S-I (Figure 1 and SI Figure S1a). This gene is associated with the synthesis of carbonic anhydrase 2, an enzyme that is involved in CaCO_3_ formation and, as a consequence, influences eggshell calcification [53]. In farm animals and poultry subject to intensive selection, especially for traits associated with growth, development, reproduction, and the composition of harvested products [110], one might expect extra selection pressure—in particular, on the expression of the *CA2* gene related to productivity in layers. In humans, the homologous *CA2* gene is also highly and, most of all, activated in the stomach, colon, and duodenum; gall bladder and liver; and, to some extent, in the appendix and small intestine [51]. At 7 dpi, the *CA2* gene regulation was highly activated in S-II and S-III relative to S-I (Figure 1 and SI Figure S1b). To the best of our knowledge, we discovered, for the first time, these effects of *Salmonella* and phytobiotic intake on the *CA2* expression in the chicken cecum, any relevant literature data being absent.

There was another interesting observation in this study regarding the *CALB1* gene which was significantly upregulated at 7 dpi in S-III relative to S-I, while its downregulated expression in S-IV at 1 dpi was restored to the level of S-I by 7 dpi (Figure 1 and SI Figure S1a,b). This may be evidence of a positive effect of the Intebio^®^ intake on the expression of this gene associated with the synthesis of calbindin, a protein involved in calcium transport, associated with the mechanical properties of eggshells in chickens [111] and slightly expressed in the human appendix [51]. The dynamics of this gene expression in response to *Salmonella* infection observed in our study on adult layers was inconsistent with the data on *CALB1* downregulation in the livers of five-month-old broilers at 10 dpi [47] and in the cecum of newly hatched layer chicks at four dpi [112], so this controversial *CALB1*-related data requires further investigation.

We reported, for the first time, the *RARRES1* gene expression in the chicken cecum. There was an upregulation of this gene at 7 dpi in response to the *Salmonella* challenge in S-II as compared with S-I (Figure 1 and SI Figure S1b). *RARRES1* is associated with the synthesis of ovocalyxin-32, a 32-kDa protein, which is part of the eggshell matrix that provides the developing embryo with protection against colonization by pathogenic microflora [54,113] and is related to the thickness of the mammillary shell layer [114]. This gene was originally identified as a target gene induced by the synthetic retinoid tazarotene (AGN 190168) in human skin wound cells [115]. There was also evidence of a surprisingly high expression of this homologous gene in the human appendix, more than in any other organ or tissue, while being also expressed in the colon, gall bladder, and small intestine [51]. RARRES1 is predicted to have metalloendopeptidase inhibitor activity [116] and was also shown to inhibit the growth of *B. subtilis*, suggesting its antimicrobial activity and contribution to the natural protection of the chick embryo [57]. In addition, the *RARRES1* gene may be a potential molecular marker associated with higher rates of egg production and affecting the performances of layers [117]. We have not found in the literature any data on the expression of the *RARRES1* gene in the chicken cecum and its differential regulation in response to infection with SE, which represents a significant novelty of our results. The new information on *RARRES1* expression in the GIT we report here, data by [51], and future studies may lead to an extended understanding and revision of the functions that this gene and its secreted product execute in the body of higher vertebrate animals and humans.

A novel finding was also obtained in our study with regards to the cecal expression of the *BPIFB3* gene encoding the synthesis of ovocalyxin-36, a 36-kDa eggshell protein. Ovocalyxin 36 is secreted in the oviducts, and its expression is usually regulated during the active calcification phase of the shell [55,118]. The *BPIFB3* expression was also found in various parts of the GIT of healthy broiler chickens, including the cecum [119]. Here, we observed a significant downregulation of this gene in the cecum of layers at 1 dpi in S-III and its upregulation in S-III and S-IV at 7 dpi (Figure 1 and SI Figure S1a,b). Considering the molecular evolution for a number of important homologous genes in amniotes, including humans, this gene was found to be highly expressed, for example, in the salivary gland at the second-highest level across various organs and tissues [51], so further investigation of the *BPIFB3* functions would be needed.

Analyzing the effect of the phytobiotic on the activation of immunity- and metabolism-related genes, we would suggest that, at 1 dpi, the adaptive response to the *Salmonella* challenge and Intebio^®^ intake in S-IV included the increased expression level of such genes as *AvBD10*, *IL6*, *IL8L1*, and *BPIFB3* as compared with S-II. Based on our results, we can also suggest that, by 7 dpi, the long-term effects of the SE challenge in S-II was likely to lead to activation of the genes *AvBD10*, *IL8L1*, *SLC5A1*, *CA2*, *CALB1*, and *RARRES1* involved in immunity, transport, metabolism, and digestion. In a few cases (SI Figure S1b), 7-dpi upregulation mediated by the SE challenge and/or supplement in S-II, S-III, and S-IV reached a factor of ~400 to ~500 (*CA2*) and even ~3100 (*BPIFB3*), which was comparable to the maximal expression of various genes in response to *Salmonella* infection in other studies [112].

### 4.2. Blood Biochemical/Immunological Variables as Affected by SE and Intebio^®^

As follows from our study results, there was a decline in the ALB content in the blood of layers at 1 dpi in both of the challenged subgroups S-II and S-IV in comparison with the nonchallenged subgroups S-I and S-III, as well as its lowest level in S-II at 7 dpi (SI Table S2). ALB is the main protein in the chicken blood serum, its fraction being the major reservoir of the protein. It also plays an important role in maintaining the colloid osmotic pressure and acid–base balance, because it works as a carrier of small molecules such as vitamins, minerals, hormones, and fatty acids [120,121]. The decreased ALB content was probably due to inflammatory process caused by *Salmonella* infection, and it was previously reported that ALB reduction (hypoalbuminemia) can be caused by GIT diseases [122].

We demonstrated, at 1 dpi, lower levels of UR and BUN in challenged hens of S-II in comparison with S-I and the appropriate increased levels in S-IV (SI Table S2), suggesting a negative effect of SE and a positive one of Intebio^®^. The CR content in the blood of all the studied subgroups was deranged and below the reference values at both 1 and 7 dpi (SI Tables S2 and S3). However, at 1 dpi, we found a greater CR level by 1.2 times in S-III as compared with S-I and by 1.3 times in S-IV relative to S-II, which may be indicative of a positive effect from the use of the additive. The fact is that CR is the end product of creatine metabolism, most CR being synthesized in the liver and transported to skeletal muscles. The CR concentration in the blood is fairly constant, reflecting the muscle mass condition, since a lower CR would be observed, along with a reduced muscle mass. Additionally, this blood biochemical index is used for the additional assessment of kidney function in poultry [120,121]. At 7 dpi, the concentrations of UR, BUN, and CR were at the same level in S-I, S-III, and S-IV, and only S-II showed a significant rise.

In our experiments, the pathological process caused by SE was accompanied by a significant growth at 7 dpi in the ALT activity in both of the challenged subgroups S-II and S-IV (SI Table S3). Moreover, it exceeded in S-IV the upper limit of the reference range for healthy birds and was 2.7 times higher than that in S-III. In clinical biochemistry, the function of transaminases ALT and AST is to catalyze the transfer of the amino group of amino acids to keto acid. In mammals, when the membranes of liver cells are disintegrated due to cell damage or metabolic changes, ALT and AST are released into the blood plasma. In general, the degree of increased ALT activity in the blood is proportional to number of damaged hepatocytes. However, these enzymes in birds are not specific for determining the liver function, and alterations in their contents do not necessarily correlate with liver damage [120,121]; therefore, the interpretation of change in avian blood enzyme activity may be different. Nonetheless, it was previously suggested that augmented ALT levels in response to *Salmonella* infection may be related to the ability of this pathogen to colonize the liver, which causes damage to its cells [59,123]. Our data on the elevated ALT levels in SE-challenged chickens was in agreement with previous observations in other studies [23,58]. Such an elevation was demonstrated in the SE-challenged subgroup (SE) of day-old ISA Brown chicks [23] at four and 18 dpi in comparison with three other subgroups: fed an EO-based sage extract (S), SE-challenged and fed the extract (SSE), and the negative control (C).

As our study demonstrated, there was a reduction of the AST content in both S-III and S-IV fed Intebio^®^ as compared with S-I and S-II at both 1 and 7 dpi, while we observed in challenged chickens at 7 dpi the maximal growth of AST in S-II, as well as a greater AST level in S-IV relative to S-III (SI Tables S2 and S3). A significant elevation in the AST activity was also shown at four dpi in ~six-month-old White Leghorn layers inoculated intramuscularly with *Salmonella* Typhimurium (ST) [124]. These findings would suggest a positive effect of phytobiotic intake and a negative one of *Salmonella* infection in the bodies of birds, taking into account that AST may be a nonspecific marker of the pathology of hepatocytes in poultry [120,121].

Combining the data on levels of UR, BUN, CR, ALT, and AST at both time points, we can see that the activity of the two aminotransferases was significantly lower at 1 dpi in the challenged S-II birds, with the concentrations of UR, BUN, and CR being also lower. However, by 7 dpi, the activity of the two enzymes, ALT and AST, in this subgroup were significantly elevated, while the levels of UR, BUN, and CR also grew. This might be indicative of an expected metabolic response to the SE challenge progression. ALT and AST mediate the involvement of amino acids into catabolic pathways and facilitate the reduction of such major protein metabolism products such as UR and CR. In other studies (e.g., in broiler chicks [125]), ALT and AST lowered their activity in response to a stress factor (e.g., a supplementation with silver nanoparticles [125]). This caused lower concentrations of UR and CR, suggesting a disturbed protein catabolism.

We found a significant elevation of ALP at 1 dpi in S-III and S-IV fed the supplement and its drop by 7 dpi in all the experimental subgroups relative to S-I (SI Tables S2 and S3). A similar lowering of ALP activity was determined at four dpi in subgroups SE, S, and SSE in comparison with C [23]. Previously, a significant decline in the ALP level was also seen in other studies, e.g., at four dpi in White Leghorn hens infected with ST [124], in cockerels in response to the Newcastle disease virus [126,127], and in broiler chicks subjected to a stress factor (silver nanoparticles [125]). However, we were unable to find any published data on the ALP activity at 1 dpi in response to *Salmonella* and/or phytobiotics, so its increase in chickens fed Intebio^®^ seems unclear, and further investigation would be wanted. Since the ALP level in S-III was at 7 dpi lower than that in S-I, this may be indicative of the protective effect of phytobiotics on tissues and parenchymal organs in healthy birds.

In both of the challenged subgroups S-II and S-IV, there was an elevation at 1 dpi in the blood levels of the digestive enzyme AMY as compared with the nonchallenged subgroups S-I and S-III, which probably reflected the activation of the carbohydrate metabolism in response to SE, taking into account that AMY is produced by the pancreas and is involved in the breakdown of starch and glycogen to GLUC. By 7 dpi, the AMY activity was reduced in S-II, which also had a declined GLUC level. A consimilar decreased GLUC concentration in response to ST was found in Leghorn fowls at four dpi [124]. In our study, there were also lower GLUC concentrations at 7 dpi in S-III and S-IV. Likewise, reduced GLUC levels were detected at four dpi in subgroups SE and SSE in comparison with C, although subgroup S showed the same GLUC content as C [23].

Challenged hens in S-II and S-IV demonstrated, at 1 dpi, a lower CHOL concentration. Similarly, a reduced CHOL level in response to a parenteral ST inoculation was identified in two broiler strains at 0.5 dpi [128] and in Leghorn chickens at four dpi [124]. Our further observations showed that, by 7 dpi, the CHOL content was restored in S-II at the negative control level or was a bit higher in S-IV.

The calcium content was found to be at lower levels in the challenged subgroups S-II and S-IV at 1 dpi, which could also be related to a downregulation of the *CALB1* gene involved in calcium transport (SI Figure S1a). At 1 dpi, the PHOS content in the blood decreased in the challenged subgroups S-II and S-IV by 1.4 and 1.6 times, respectively, as compared with the nonchallenged subgroups S-I and S-III, and it was lower in S-II by 7 dpi, as well. This seems to be quite natural, since a decline in the PHOS content in birds is usually observed with loss of appetite or bowel disease [120,121].

A dropped BIL concentration was previously seen in subgroups SE, S, and SSE at four dpi and its elevated levels in the same subgroups at 18 dpi relative to C [23]. We found a different pattern of changes in the BIL amount, though at two different time points, as follows: a downswing in S-II and growth in S-III and S-IV at 1 dpi and decline in S-III and increase in S-IV at 7 dpi. The effects of SE and EOs on the BIL levels in the chicken body would require further examination.

Unlike mammals that produce UR as a result of the breakdown of amino acids, the main product of the metabolism of nitrogen-containing compounds in birds is URA. We identified that there was an abrupt fall in the URA content at 1 and 7 dpi in both of the challenged subgroups S-II and S-IV relative to the nonchallenged subgroups S-I and S-III. This observed phenomenon is consistent with other studies showing that a protein deficit in the diet leads to a diminution in the serum URA concentration [120,121]. Probably, a *Salmonella* infection in chickens may cause a reduction in protein digestibility and absorption.

The SLA usually declined in challenged poultry [129,130,131,132], although we determined an opposite effect at the two time points in S-II, S-III, and S-IV, suggesting a need for further exploration of the SLA in chickens infected with SE and/or fed EOs.

There was a 1.5-fold decline in the SBA level in S-II as compared with S-I, while no SBA decrease was identified in S-IV. This might give evidence of a positive effect of the phytobiotic intake in birds, since SBA is an integrative factor for the natural resistance of the humoral type, suggesting an ability of the blood to “self-clean”. A drop in the SBA index may be due to a profound impairment of the immune system and serves as an unfavorable prognostic indicator [120,121]. Based on the Pearson’s correlation coefficient test, we identified a significant direct relationship between the *IL6* gene expression and SBA in hens at 1 and 7 dpi.

The correlation analysis also showed a significant direct relationship of the gene expression of *AvBD10* (at 1 dpi) and *IL6* (at 7 dpi) with BLA. This correlation between BLA and the expression of immunity/inflammation-related genes seems to be meaningful, since an increase in the level of β-lysines, cationic serum proteins produced by platelets and demonstrating bactericidal activity against aerobic spore-forming bacteria, is usually revealed in the acute phase of inflammatory processes [120,121].

In the challenged subgroups S-II and S-IV, we observed, at 1 dpi, a lower content of all the studied immunoglobulin classes: IgA, IgM, IgG1, and IgG2, which are glycoproteins playing an important role in the functioning of innate immunity and produced in response to an exposure to antigens of bacteria, viruses, micromycetes, protozoa, etc. At 7 dpi, there was a rise in the levels of IgM and IgG1 in S-IV as compared with S-III. Interestingly, the correlation analysis revealed the presence of a significant direct relationship between the *IL8L1* gene expression and IgA content at 1 dpi and between the gene expression of *IL6* and *IL8L1* and IgM activity.

If we examined an entire set of the biochemical/immunological indices, the challenged S-II subgroup was, at 1 dpi, the most different from the other subgroups, whereas S-IV was closer to S-III, forming a cluster of these two subgroups fed Intebio^®^, which was then combined with S-I (SI Figure S2a,e), suggesting a positive effect of Intebio^®^ administration at the acute SE challenge phase. The further progression of immune and metabolic responses to *Salmonella* led, at 7 dpi, to somewhat similar blood biochemical/immunological changes in the challenged and nonchallenged subgroup clusters (SI Figure S2b,f).

Thus, the assessment of blood biochemical/immunological parameters characterizing the levels of the metabolic processes and adaptive metabolic homeostasis in laying hens suggested significant negative shifts in the metabolism due to SE challenge and a positive effect of the administration of the phytobiotic Intebio^®^. The expression levels of the genes associated with immunity correlated with some biochemical/immunological blood parameters that reflect the activation of the chicken immune system.

### 4.3. Effects of SE and Intebio^®^ on Cecal Microbiome Profiles

Judging from the alpha biodiversity parameters of the cecal microbial communities (Table 1), the SE challenge and/or Intebio^®^ intake did not affect the number of OTUs in any of the subgroups within one time point. No differences were also demonstrated in the total number of OTUs when comparing the microbiota profiles in the cecum of male ISA Brown chicks infected with SE after being hatched vs. nonchallenged control birds [133]. However, for assessing the biodiversity of communities, one should take into account not only such a descriptive metric as the number of species (OTUs) but, also, their relative abundance or “evenness”. The Shannon diversity index takes into account both the species richness and “uniformity” of OTUs [134], and, as noted in Table 1, there was a slight decrease in the Shannon index at 1 dpi in S-IV as compared with S-I, meaning some lowering in the biodiversity [135]. Thus, the simultaneous SE challenge and phytobiotic administration had some impact on the relative abundance and “evenness” of the species but not on their number.

The comparison of the beta diversity in the cecal microbiota of laying hens from the four subgroups using the PCA showed that, at 1 dpi, the individual microbiome profiles in S-IV, as well as, to some extent, those in S-II, were separated into distinct clusters, suggesting a certain difference in the structure of microflora in these subgroups relative to the nonchallenged S-I and S-III hens (SI Figure S3a). Interestingly, the greatest displacement along the axis of the first principal component (PC1) was shown for the S-IV samples, whereas the other subgroups gravitated towards the PC2 axis. This would additionally provide evidence of a relatively unique microbiome composition in S-IV. Additionally, S-III and S-IV had a greater microbiome variability than the two other subgroups, meaning that the greatest homogeneity within a subgroup was observed among the samples of S-I and S-II. Moreover, the composition of individual microbiomes in the nonchallenged S-III had more affinity with S-I. By 7 dpi, a definite change occurred in the subgroup clustering pattern, reflecting a shift in their microbiota profiles (SI Figure S3b). These results demonstrated a definite effect of the *Salmonella* infection on the alterations in the composition of the cecal microbiome in adult laying hens from 1 dpi to 7 dpi. Previously, in the study of bacterial profiles in male chicks reared for a few weeks after hatching [133], there was no obvious PCA-based clustering of SE-challenged and nonchallenged groups, as well as turns in the microbiota profiles after infection, suggesting that those data [133] are not directly comparable to our study.

In addition, we produced Ward’s clustering of the cecal microbiome profiles at the two time points (Figure 2 and SI Figure S4). At 1 dpi, the microbiomes in the nonchallenged subgroups S-I and S-III merged into a common cluster that was then joined by S-II, with the inter-subgroup distances being very small (Figure 2a and SI Figure S4). This could illustrate small differences in the bacterial profiles between the nonchallenged subgroups S-I and S-III, on the one hand, and the SE-challenged S-II, on the other, suggesting that 1 dpi would not be a sufficient period for *Salmonella* in S-II to develop a pronounced effect on the microflora composition. In contrast, S-IV subjected to two factors (Intebio^®^ and SE) stood out separately and at a considerable distance from the other subgroups. Thus, judging from the results of both PCA and Ward’s clustering, the combination of the two factors in S-IV had, at 1 dpi, a pronounced effect on its cecal microbiota profile. By 7 dpi, the tree topology essentially altered, reflecting the progression of SE infection that caused a more evident and quite similar shift in the microflora profiles in S-II and S-IV in comparison with the cluster of nonchallenged subgroups S-I and S-III (Figure 2b and SI Figure S4).

We suggest that the changes we observed in the profiles of the intestinal microbiota in chickens are self-consistent, since it is known [134,135] that the infection of the organism with *S. enterica* leads to an active synthesis of antimicrobial metabolites in the intestines, such as proteases, reactive oxygen species, nitric oxide radicals, and chelators. Many of these antimicrobial agents can cause a reduction in the number of certain microorganisms.

As a result of assessing the diversity of the metagenomic community in the cecum of laying hens at the level of orders and higher taxonomic ranks (Figure 3), we detected 13 phyla of attributable microorganisms. The microflora at the phylum level was dominated by Firmicutes and Bacteroidetes. Previously, a 16S rRNA gene sequencing-based study showed [136] that representatives of the phyla Firmicutes and Proteobacteria accounted for more than 90% of the analyzed sequences in male chicks infected with ST at four days old, which might differ from our results. However, differences in the compared profiles of the intestinal microbiome could be associated with different ages in the studied birds, as well as with different diet compositions and the use of antibacterial drugs. Specifically, a certain selective effect of the antibiotic virginiamycin used in the compound feed (SI File S1) on the presence of some representatives in the microflora might be seen in our experiment, suggesting a competitive exclusion of some microorganisms and a boosted colonization with the others. In our opinion, the prevalence of Firmicutes and Bacteroidetes bacteria in the microbiome that we showed here can be explained by the fact that these taxa mainly include anaerobic forms, and their abundance is self-consistent due to a lower redox potential in the intestine.

In our study, a significant proportion among the Firmicutes phylum were the order Clostridiales bacteria (40.3% ± 3.61% to 52.0% ± 4.39%). The *Salmonella* challenge of chickens in S-II did not significantly affect the number of these microorganisms, whereas an evident increase in the bacterial content of the order of Clostridiales was observed in S-IV exposed to SE and the phytobiotic as compared with S-I. A significant abundance of these microorganisms in the lumen of chicken intestines was previously reported [137]. Pathogenic bacteria such as *Clostridium tetani* (tetanus agent), *C. botulinum* (botulism pathogen), and *C. perfringens* (gas gangrene agent) are often found among the order Clostridiales bacteria [138]. Additionally, among the order Clostridiales representatives, there are many microorganisms (families Lachnospiraceae, Eubacteriaceae, Ruminococcaceae, etc.) that produce cellulase during the metabolism [139]. This enzyme catalyzes the hydrolysis of β-1,4-glycosidic bonds in cellulose delivered with feed, with the formation of GLUC or cellobiose, taking an active part in the digestion process. In our experiments, there was no significant elevation in S-IV (as compared with S-I) in the content of the genus *Clostridium* bacteria—among which, pathogenic forms are often present. In this regard, an uplift in other bacteria from the order Clostridiales in S-IV may suggest a positive role of the phytobiotic in restoring the normal composition of the intestinal microbiome.

Among the phylum Bacteroidetes microorganisms, the order Bacteroidales bacteria were abundant in the chicken intestines in all subgroups (38.3% ± 3.85% to 48.3% ± 3.25%), with a rise in the number of these microorganisms in S-II and S-III relative to S-I and the decreased contents of these bacteria in S-IV. The Bacteroidales order microorganisms uncultivated on nutrient media were increasingly recognized as the predominant representatives of the intestinal microbiota in chickens [140,141]; however, the nature of their interactions with the host in birds has not been fully uncovered. In mice and humans, it was shown [142] that these microorganisms can be both commensals and pathogens. It is known [143] that they play the role of symbionts in the GIT of humans, animals, and birds, metabolizing carbohydrates, including starch, to acetate, succinate, and isovalerate. It has been repeatedly noted that some representatives of this taxon, e.g., *Bacteroides fragilis*, are often the predominant microorganisms in polymicrobial infections of humans. Moreover, the synthesis of β-lactamase by some of them contributes to an increased antibiotic resistance to β-lactam antibiotics. Bacteremia caused by *B. fragilis* has been reported, with an associated human mortality rate of 27%. Many bacteria of the Bacteroidales order [142] have a SpeB homolog, a peptidase capable of cleaving many immunologically relevant proteins. In particular, the periodontal pathogen *Prevotella intermedia* has a homolog interpain A that takes part in the inhibition of the immune response through complement degradation [144].

A peculiar and controversially abruptly fall in number of the genus *Bifidobacterium* representatives (among which, *B. longum* dominated) (SI Figure S5a) in the challenged subgroups S-IV (by 12.5 times) and S-II (by 10.5 times) relative to S-I. The representatives of this taxon have a number of useful properties for the body, including antagonistic properties against pathogens, the synthesis of water-soluble vitamins, the digestion of plant oligo- and polysaccharides, the suppression of the production of potentially toxic metabolites, and the modulation of the host immunity [145]. In this regard, the decline in the number of Bifidobacteria in the challenged subgroups may be indicative of dysbiotic disorders in the composition of the intestinal microflora.

We also detected a similar pattern of a reduced content of the order Lactobacillales representatives (mainly, the genus *Lactobacillus* microorganisms) in S-IV in comparison with S-I. Many bacteria of this taxon produce bacteriostatic and bactericidal compounds like organic acids, bacteriocins, and others [146], inhibiting microbes that can be pathogenic to chickens, such as *Escherichia coli*, *Salmonella* spp. [147], *Clostridium perfringens* [148], *Campylobacter jejuni* [149], and others. Our data on a reduction in the number of the order Lactobacillales representatives due to the infection of chickens with *Salmonella* contradict a certain other study [133] on infecting chickens with SE, which showed no significant changes in the number of microorganisms, with the exception of the Lactobacillaceae family representatives, the contents of which, in contrast to our results, grew.

Interestingly, there was a lessening in the content of methanogenic archaea of the genus *Methanobrevibacter* in the cecum of hens in S-IV (by 1.9 times) and S-II (by 1.2 times) as compared with S-I (SI Figure S5a). The presence of methanogenic archaea in the GIT lumen of birds is known [150], but their ecological role in the body of birds has not been studied. In the example of cattle, whose methanogenic symbionts play a significant role in environmental pollution, it was shown that, in the rumen, these microorganisms exclusively use H_2_, CO_2_, and, to a lesser extent, formate as methanogenic precursors, despite the fact that methanol; acetate; and mono-, di-, and trimethylamines also represent potential substrates for fermentation [151]. Hydrogen removal from the rumen occurs mainly as a result of methane formation reactions, the latter being the only energy-generating process in these bacteria that requires the presence of cofactors [152]. It was found that the transformation of carbon dioxide to CH_4_ is of great importance for the effective course of fermentation in the rumen, since it prevents the accumulation of reducing equivalents [153]. However, this process causes significant environmental damage, since, on average, about 20% of the total methane emissions into the atmosphere are due to methanogenesis in the digestive tract of ruminants [154].

It was previously demonstrated that, in the rumen of cattle, amylolytic bacteria synthesizing propionate are able to compete with methanogens for the use of hydrogen [155]. In our experiment, a lowered content of methanogenic archaea of the genus *Methanobrevibacter* in the chicken cecum in S-II and S-IV could be associated with an elevation in the number of amylolytic microorganisms of the order of Bacteroidales due to competitive exclusion.

Among the order Bacteroidales bacteria, the most pronounced variation in the amount was found between the subgroups for the dominant species *Bacteroides barnesiae* (SI Figure S5a). This species is a typical representative of the order Bacteroidales and a common inhabitant of the intestinal lumen of mammals and birds [156].

In our research, an increase in the content of *Campylobacter* bacteria was noted in S-IV (by 5.6 times) and S-II (by 5.5 times) relative to S-I, which may be associated with a reduced amount of the order Lactobacillales representatives that synthesize antimicrobial substances. This could be indicative of dysbiotic disorders in the intestinal microbiome as a result of an infection with SE, since pathogens such as *Campylobacter jejuni* and *E. coli* are often found among bacteria of the genus. Despite the fact that birds, as a rule, show resistance to infection by these pathogens, some representatives of *Campylobacter* spp. are the main cause of foodborne gastroenteritis in humans [157].

In addition, among the microflora we examined in the cecum of hens, unidentified candidate division TM7 bacteria (up to 0.29% ± 0.02%) and candidate division WPS-2 (phylum) (up to 1.4% ± 0.06%) were found in very small quantities. There are studies showing the presence of the candidate division TM7 bacteria in the contents of the GIT of birds [158], including chickens [159]. However, the role of these microorganisms in the intestines of birds was unclear. A relationship was found between the increased amount of TM7 bacteria in the human intestine and inflammatory bowel diseases [160], which may suggest their negative role in the host. As for the WPS-2 microorganisms, the presence of these bacteria was previously observed in the feces of *Strigops habroptila* parrots [161]. In the lumen of the chicken cecum, these microorganisms were discovered by us for the first time. The role of these bacteria has not yet been disclosed. In our experiment, there was a significant decline in the contents of these microorganisms in S-II and S-III, whereas their numbers remained unchanged in S-IV relative to S-I.

Overall, we can suggest that, although we did not observe any significant direct effects of Intebio^®^ on preventing or reducing the infection of *Salmonella*, the diet supplementation with the phytobiotic could be related to changes in the whole microbiota profile and separate microbial taxa.

### 4.4. Egg Production Traits and Effects of SE and Intebio^®^

One of the major aims of the study was to identify differences between the subgroups (control and experimental ones) in the mean egg weight and other performance traits during the experiment period, both before and after the challenge with SE. Herewith, the physiological period should be taken into account, i.e., the peak of egg production that the chickens were at. During this period, the biological limit of productivity is observed in birds; therefore, the information obtained in the experiment made it possible to track the effectiveness of the phytobiotic and the effect of the SE challenge on these traits.

Over a 27-day period of the experiment, we revealed significant differences between the subgroups (control and experimental ones) in the mean egg weight and other egg productivity traits.

The minimum rate of decline in egg production was observed in S-III layers fed the Intebio^®^ additive, suggesting that the phytobiotic properties had a positive effect on their performance. Obviously, the use of Intebio^®^ also had a certain positive effect in S-IV hens infected with *Salmonella* and fed the supplement. Due to slowing down the decreasing rate in the egg production function, the productivity of chickens in this subgroup leveled out in relation to the control (S-I) by the end of the 27-day laying period (Figure SI2-1d).

Overall, significant differences were found between the experimental subgroups in terms of egg productivity—specifically, egg weight—with a negative effect of infection in the SE-challenged hens and a positive one when adding phytobiotics to the chicken diet.

### 4.5. Interplay between Treatments and Studied Characters

Using the PCA technique (SI Figure S6 and Figure 4), we established in this study on SE infection and supplement of the chicken diet with phytobiotics that the main significant determinants were such biochemical/immunological blood factors as the content of serums ALB, PHOS, UR, BUN, BIL, CR, ALT, SBA, SLA, and BLA and immunoglobulins (IgA, IgM, IgG1, and IgG2), as well as egg production traits. Probably, their observed variations reflected the processes due to SE infestation to the greatest extent, suggesting that these factors responded to infection at a faster rate. The phytobiotic administration and SE challenge had a strong influence on the formation of individual clusters in two-dimensional PCA graphs using a complex of the studied characters (SI Figure S6).

Thus, by applying a PCA, it was feasible to visualize the acute phase of the pathological process at 1 dpi and its further progression by 7 dpi and reveal the distinctive factors affecting S-III and S-IV fed the phytobiotic supplement.

The set of determinants we considered in the course of the PCA analysis and identified as the significant ones confirmed our assumption about the positive effects of the phytobiotic treatment on the layers. Egg productivity was found to be closely linked to the biochemical/immunological blood composition. The infestation with the pathogen affected all systems of the chicken body.

The results of the Ward’s clustering-based analysis of the microflora composition (Figure 2 and SI Figure S4), as well as the implementation of the PCA method using the complex of all the studied characteristics (SI Figure S6 and Figure 4), showed that the arrangement of the subgroups in distinct clusters could occur due to the two key treatment factors, i.e., SE infection and the phytobiotic supplement. Each produced cluster was described by a number of unique characteristics. Judging from the comprehensive correlation study, a change in the level of *IL8L1* gene expression positively correlated with a change in the level of IgA activity, for the genes *IL6* and *IL8L1* with IgM activity, and for the genes *AvBD10* and *IL6* with BLA. Regardless of the use of the phytobiotic, there was a decline in the levels of IgA, IgM, IgG1, and IgG2 at 1 dpi. Thus, the observed clusters of the studied subgroups were dynamically dependent on the time factor associated with the development of the infestation process and the immune response of the bird’s body. At 1 dpi, the key biochemical/immunological blood factors were the serum levels of IgA, IgM, IgG1, IgG2, ALB, PHOS, URA, BLA, SLA, BIL, and CR. At 7 dpi, the other factors became important, including the contents of UR, BUN, CR, SBA, ALT, BIL, IgM, ALP, and SLA. The phytobiotic effect on the factors of the immune response could be seen, such that the IgG1 level was elevated by 7 dpi in S-IV, and the level of CR in the blood was higher in S-III and S-IV.

We also looked closer into the IgM changes by applying the multicharacter model (1). These changes, at first glance, might reflect the development of an immune response by 7 dpi. However, as demonstrated in Formula (1), the coefficient for the Intebio^®^ administration factor was 0.624 or almost 1.8 times higher than that for the SE infection factor (0.350), and this may suggest a greater contribution of Intebio^®^ to the IgM level than that of SE infection. Additionally, we determined that a significantly close correlation between CHOL and IgM is consistent with the data on the relationship of CHOL with IgM and IgG in the serum found by other authors [162], while the CHOL level was linked to other serum biochemical indicators and the visceral fat of chickens, considering breed and gender differences as important factors for variations in the blood serum parameters [163].

## 5. Conclusions

Our multifaceted investigations suggest that the challenge with SE and the administration of the phytobiotic Intebio^®^ based on essential oils in the diet of laying hens had both a depressing and an activating effect on the expression of certain studied genes and performances. DGE was observed at both 1 and 7 dpi for genes associated with immunity, the transport of molecules/ions, and the metabolism in the intestine. We also suggested parallels with the mammal/human tissue-specific expression of homologous genes as evidence of existing evolutionary conservation in gene functions between remote higher vertebrate taxa.

We observed the effects of the pathogen infection and phytobiotic supplementation on the microbiome profiles in the cecal contents and biochemical/immunological blood parameters, including such indicators of the immune system activity of birds as the level of SBA, BLA, and immunoglobulins. As we showed, the SE challenge negatively affected the state of the GIT microbiome and the level of the metabolism in hens, whereas the Intebio^®^ intake in the infected hens could cause a pronounced shift in the microbiota composition in the cecum at 1 dpi. The supplementation of the layer diet with the phytobiotic suggested a certain positive effect on the state of the body’s metabolic activity and an elevation of their adaptive potential reflected by the activated expression of some genes in both challenged and nonchallenged birds. Significant differences were found between the subgroups in terms of the egg production traits, with a negative effect of SE infection on them in the challenged subgroup and a positive effect of the phytobiotic specifically on egg weight.

Judging from a comprehensive assessment of the produced data, the tested feed additive was not toxic, while having the potential to create a certain mobilizing status of the immune system due to the activation of a number of responsible genes, probably, even before infection. The supplemental effects were also reflected in the changes in the biochemical/immunological blood indices and cecal microflora profiles and, as a consequence, in maintaining the egg production traits at the levels of healthy layers.

## Figures and Tables

**Table 1:** Estimates of the alpha diversity parameters (M ± m).

Subgroups	OTUs		Shannon Index	
	1 dpi ^1^	7 dpi ^1^	1 dpi	7 dpi
S-I	601.7 ± 44.8 ^a^	385.7 ± 56.6 ^a^	6.6 ± 0.02 ^a^	6.4 ± 0.29 ^a^
S-II	610.7 ± 35.8 ^a^	428.3 ± 49.1 ^a^	6.1 ± 0.39 ^a,b^	6.5 ± 0.06 ^a^
S-III	570.0 ± 25.2 ^a^	390.7 ± 36.3 ^a^	6.3 ± 0.13 ^a,b^	6.3 ± 0.22 ^a^
S-IV	521.3 ± 19.7 ^a^	435.3 ± 16.2 ^a^	6.1 ± 0.07 ^b^	6.4 ± 0.21 ^a^

^1^ Significant differences between operational taxonomic units (OTUs) at 1 vs. 7 days post-inoculation (dpi) within each subgroup (*p* < 0.05). Subgroups: S-I (negative control), S-II (*Salmonella enterica* serovar Enteritidis (SE) challenge), S-III (Intebio^®^ intake), and S-IV (Intebio^®^ intake + SE challenge). ^a,b^ Data within one column with no common letters differed significantly (at *p* < 0.01). OTUs: operational taxonomic units.

**Table 2:** Mean values of egg production traits by subgroup (M ± m).

Subgroups ^1^	Egg Weight, g	No. of Laid Eggs	Egg Mass, g
S-I	64.94 ± 1.91 ^a^	8.44 ± 1.76 ^a^	547.54 ± 111.03 ^a^
S-II	64.29 ± 2.43 ^a,c^	7.48 ± 2.10 ^a,b^	479.19 ± 134.37 ^b^
S-III	66.10 ± 2.19 ^b,c^	8.56 ± 1.40 ^a,c^	564.81 ± 91.2 ^a,c^
S-IV	66.33 ± 1.71 ^b^	7.52 ± 2.10 ^a,b^	498.86 ± 141.17 ^a,b^

^1^ Subgroups: S-I (negative control), S-II (SE challenge), S-III (Intebio^®^ intake), and S-IV (Intebio^®^ intake + SE challenge). ^a–c^ Data within one column with no common letters differed significantly (at *p* < 0.01).

## Data Availability

Data is contained within the article or supplementary material.

## Supplementary Materials

The following are available online at https://www.mdpi.com/2076-2615/11/2/360/s1: SI Table S1: Gene-specific primers for assessing the expression of eight chicken genes. SI Table S2: Blood biochemical/immunological parameters in laying hens at 1 dpi. SI Table S3: Blood biochemical/immunological parameters in laying hens at 7 dpi. SI Figure S1: Relative quantification (RQ) of the expression of the eight genes associated with immunity, transport, and metabolism in laying hens at 1 (a) and 7 dpi (b) in the subgroups. SI Figure S2: Subgroup clustering based on the blood biochemical/immunological indices at 1 (a,c,e) and 7 dpi (b,d,f). SI Figure S3: Principal component analysis of the microbial diversity in samples represented as three-dimensional EMPeror graphs using the Weighted UniFrac metric (one point corresponds to one bird) at 1 (a) and 7 dpi (b) in the subgroups. SI Figure S4: Subgroup clustering based on the chicken cecal microbiomes at 1 and 7 dpi. SI Figure S5: Cecal microbiome biodiversity of laying hens at 1 (a) and 7 dpi (b) at the lower taxonomic levels. SI Figure S6: A generalized analysis of the subgroup samples by a complex of the measured traits at 1 (a,b) and 7 dpi (c,d). SI File S1: Chicken housing and management conditions. SI File S2: Effects of the SE challenge and Intebio^®^ intake on egg production.
