# Supplementary material for: Effects of Essential Oils-Based Supplement and Salmonella Infection on Gene Expression, Blood Parameters, Cecal Microbiome, and Egg Production in Laying Hens
Source: Animals (Basel). 2021 Feb 1;11(2):360. doi: 10.3390/ani11020360 (PMC7912222; doi:10.3390/ani11020360)
Supplement: Supplementary file 1 [file animals-11-00360-s001.zip › SuppInfo Figure S1.docx]

| 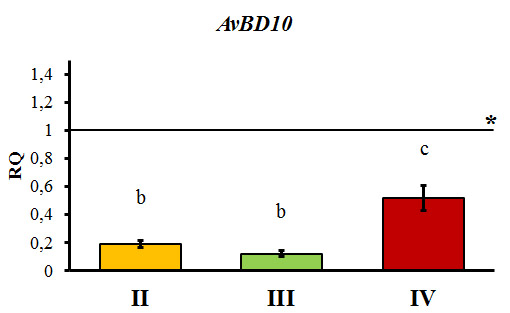 | 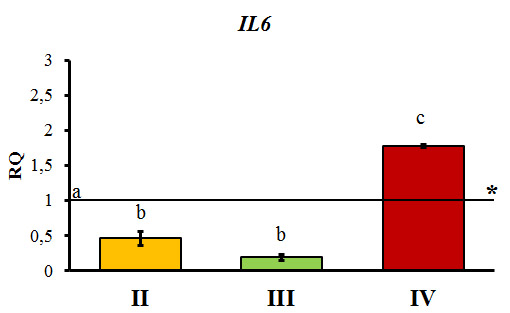 |
| --- | --- |
| 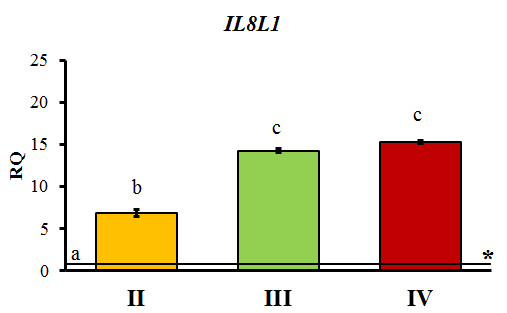 | 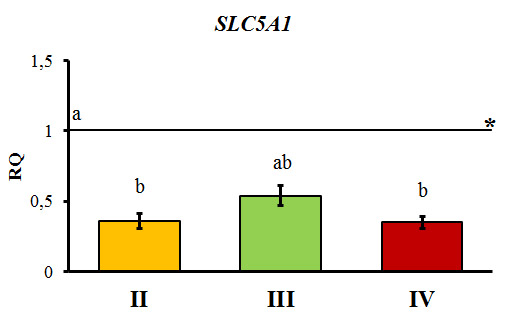 |
| 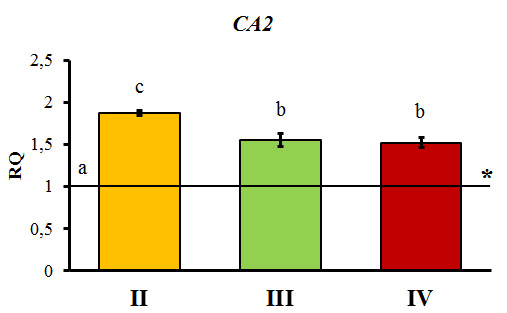 | 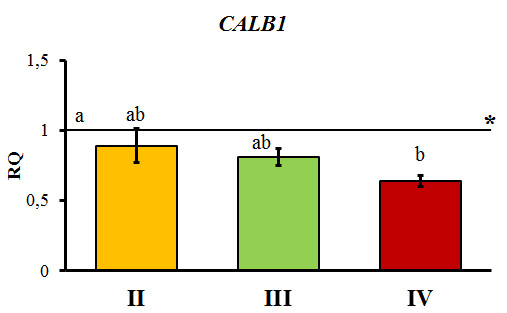 |
| 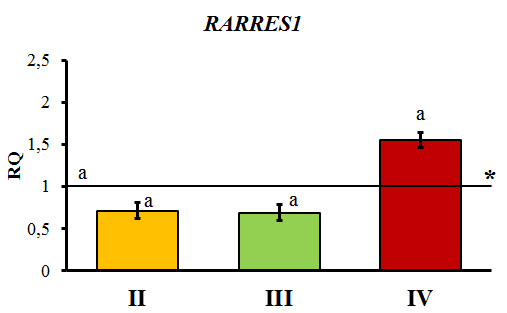 | 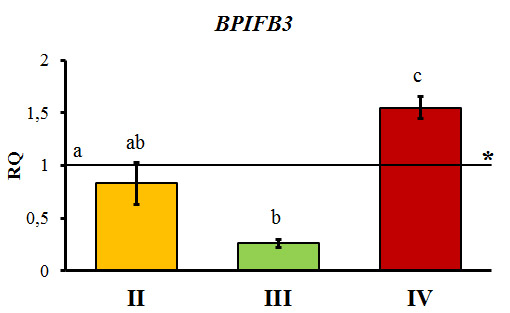 |

**(a)**

| 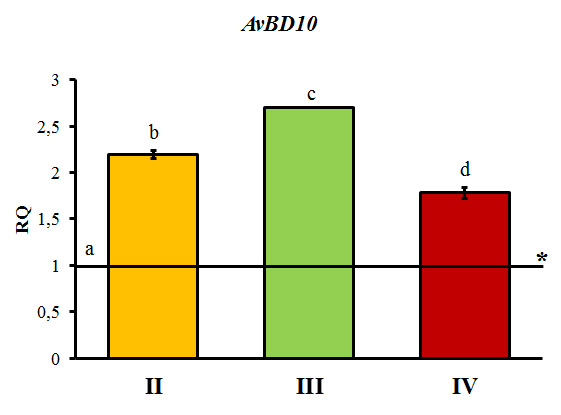 | 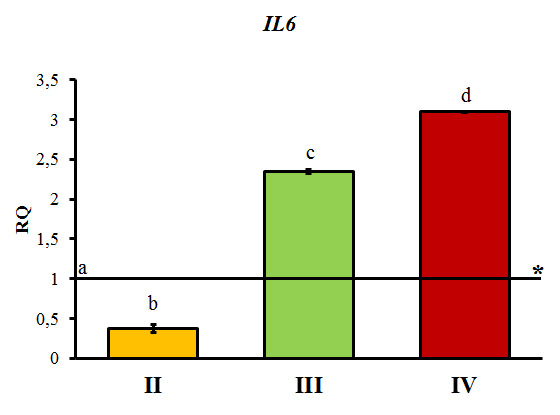 |
| --- | --- |
| 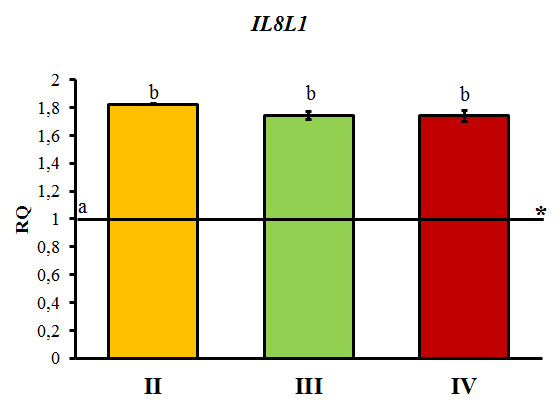 | 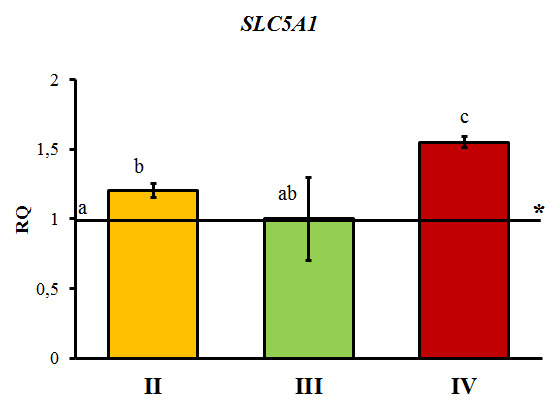 |
| 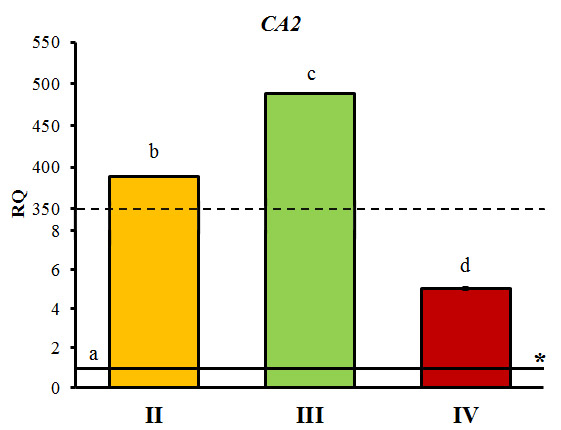 | 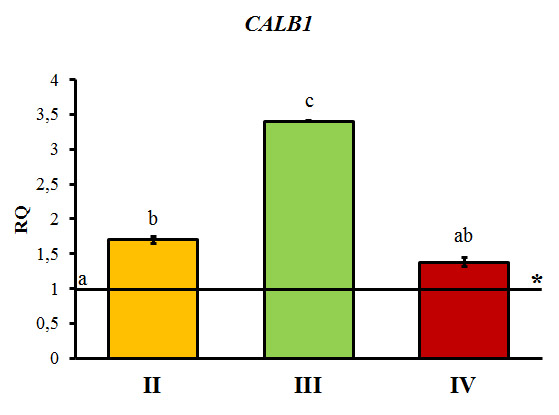 |
| 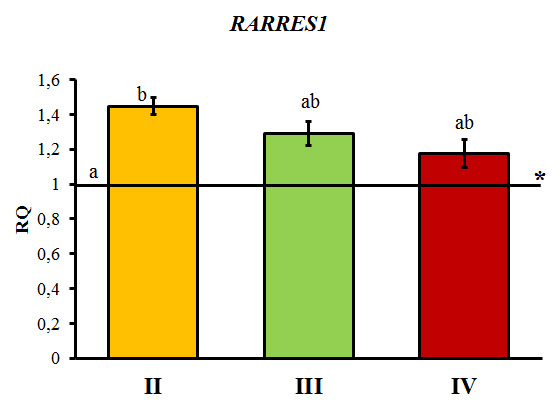 | 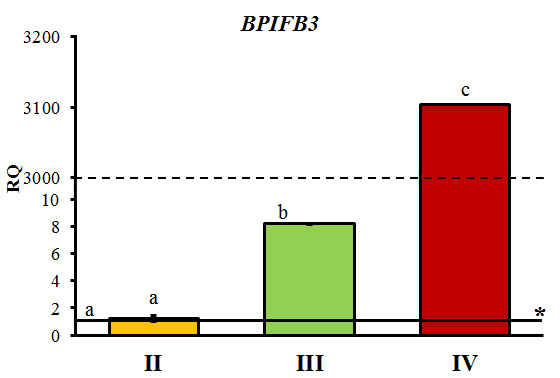  **(b)** |

**Figure S1.** Relative quantification (RQ) of expression of the eight genes associated with immunity, transport and metabolism in laying hens at 1 (a) and 7 dpi (b) in the subgroups: I (negative control; its relative basal expression level is taken as 1 and is shown by a solid horizontal line marked with *), II (SE challenge), III (Intebiio intake), IV (Intebio intake + SE challenge). ^a–c^ Mean RQ values for the control and experiment subgroups within a gene with no common letters differed significantly (*p* < 0.05), with the letter ‘a’ corresponding to Subgroup I.
