# Supplementary material for: Effects of Essential Oils-Based Supplement and Salmonella Infection on Gene Expression, Blood Parameters, Cecal Microbiome, and Egg Production in Laying Hens
Source: Animals (Basel). 2021 Feb 1;11(2):360. doi: 10.3390/ani11020360 (PMC7912222; doi:10.3390/ani11020360)
Supplement: Supplementary file 1 [file animals-11-00360-s001.zip › SuppInfo Figure S2 3.docx]

**(a)**  (**b)**

**(c)**  (**d)**

**(e)**  (**f)**

**Figure S2.** Subgroup clustering based on the blood biochemical/immunological indices at 1 (a, c, e) and 7 dpi (b, d, f). For reconstructing the trees (a), (b), (c) and (d), the Ward’s hierarchical agglomerative clustering was applied using a matrix of squared Euclidean distances between objects, with the data being normalised for the trees (c) and (d). For producing the trees (e) and (f), the UPGMA method was employed using Euclidean distances. Bootstrapping validation was performed using AU (Approximately Unbiased) *p*-values (%) and BP (Bootstrap Probability) values (%) shown with red and green estimates, respectively. Clusters with AU *p*-values greater than 95% are placed within red rectangles. Subgroups: I (negative control), II (SE challenge), III (Intebio intake), IV (Intebio intake + SE challenge).
