# Supplementary material for: Effects of Essential Oils-Based Supplement and Salmonella Infection on Gene Expression, Blood Parameters, Cecal Microbiome, and Egg Production in Laying Hens
Source: Animals (Basel). 2021 Feb 1;11(2):360. doi: 10.3390/ani11020360 (PMC7912222; doi:10.3390/ani11020360)
Supplement: Supplementary file 1 [file animals-11-00360-s001.zip › SuppInfo Figure S3.docx]

**a**  **b**

**Figure S3.** Principal components analysis of microbial diversity in samples represented as three-dimensional EMPeror graphs using the Weighted UniFrac metric (one point corresponds to one bird) at 1 (a) and 7 dpi (b) in subgroups: I (negative control), II (SE challenge), III (Intebio intake), IV (Intebio intake + SE challenge).
