# Supplementary material for: Effects of Essential Oils-Based Supplement and Salmonella Infection on Gene Expression, Blood Parameters, Cecal Microbiome, and Egg Production in Laying Hens
Source: Animals (Basel). 2021 Feb 1;11(2):360. doi: 10.3390/ani11020360 (PMC7912222; doi:10.3390/ani11020360)
Supplement: Supplementary file 1 [file animals-11-00360-s001.zip › SuppInfo Figure S4 4.docx]

**Figure S4.** Subgroup clustering based on the chicken caecal microbiomes at 1 and 7 dpi. For the tree construction, the Ward’s hierarchical agglomerative clustering was applied using a matrix of squared Euclidean distances between objects. Bootstrapping validation was performed using AU (Approximately Unbiased) *p*-values (%) and BP (Bootstrap Probability) values (%) shown with red and green estimates, respectively. Clusters with AU *p*-values greater than 95% are placed within red rectangles. Four clusters in the first tree (a) that were formed at lowest distances (< 5) are represented below the blue line. Subgroups: I (negative control), II (SE challenge), III (Intebio intake), IV (Intebio intake + SE challenge).
