# Supplementary material for: Effects of Essential Oils-Based Supplement and Salmonella Infection on Gene Expression, Blood Parameters, Cecal Microbiome, and Egg Production in Laying Hens
Source: Animals (Basel). 2021 Feb 1;11(2):360. doi: 10.3390/ani11020360 (PMC7912222; doi:10.3390/ani11020360)
Supplement: Supplementary file 1 [file animals-11-00360-s001.zip › SuppInfo Figure S6 2.docx]

**(a)** (**b)**

**(c)** (**d)**

**Figure S6.** A generalized analysis of subgroup samples by a complex of measured traits at 1 (a, b) and 7 dpi (c, d) using the PCA method and two-dimensional PCA graphs (one point corresponds to one bird; average values for a subgroup are represented by a point of changed colour and larger diameter) for the coordinates of the principal components: PC1 and PC2 (a, c), and PC1 and PC3 (b, d). Subgroups: I (negative control), II (SE challenge), III (Intebio intake), IV (Intebio intake + SE challenge). Eight genes tested for expression were *AvBD10*, *IL6*, *IL8L1*, *SLC5A1*, *CA2*, *CALB1*, *RARRES1* and *BPIFB3* (see their full names and detailed information in Supporting Information Table S1). Blood biochemical/immunological parameters: total protein (TP), albumins (ALB), globulins (GLOB), urea (UR), urea nitrogen (BUN), creatinine (CR), alanine aminotransferase (ALT), aspartate aminotransferase (AST), alkaline phosphatase (ALP), alpha amylase (AMY), glucose (GLUC), total cholesterol (CHOL), total calcium (CaT), phosphorus (PHOS), bilirubin (BIL), uric acid (URA), lysozyme activity (SLA), bactericidal activity (SBA), β-lysine activity (BLA), immunoglobulins (IgA, IgG1, IgG2, IgM). Egg productivity traits included mean egg weight (egg_w), number of laid eggs (egg_N) and egg mass (egg_W), and were recorded for 3 days at the first time point (at 1, 2 and 3 dpi) and at the second time point (at 5, 6 and 7 dpi).
