# Supplementary material for: Effects of Essential Oils-Based Supplement and Salmonella Infection on Gene Expression, Blood Parameters, Cecal Microbiome, and Egg Production in Laying Hens
Source: Animals (Basel). 2021 Feb 1;11(2):360. doi: 10.3390/ani11020360 (PMC7912222; doi:10.3390/ani11020360)
Supplement: Supplementary file 1 [file animals-11-00360-s001.zip › SuppInfo File S1.docx]

**Supporting Information File SI1**

*Chicken Housing and Management Conditions*

During the experiments, the requirements and recommendations for housing, keeping and feeding of the Lohmann White hybrid laying hens were abided, including the norms of the Federal Scientific Centre “All-Russian Poultry Research and Technological Institute” and the regulations of the [European Convention for the Protection of Vertebrate Animals used for Experimental and other Scientific Purposes, ETS No 123](https://rm.coe.int/168007a67b) (Strasbourg, 18 March 1986). All birds were euthanized in accordance with Protocol No. 15-065-A approved by the Institutional Committee for the Care and Use of Animals (OLAW No. D16-00214).

For the experiment, fertile hatching eggs were obtained from a private enterprise, JSC Sinyavinskaya Poultry Farm (Priladozhsky, Russia). Incubation was carried out in the hatchery of the All-Russian Veterinary Research Institute of Poultry Science using a Stimul IP-16 incubator (Stimul-Ink, Pushkino, Russia) and following the Lohmann White recommendations. Weak and sick chickens were culled prior to the study, regardless of their weight.

During the experiment, birds were kept in cage batteries (Big Dutchman Int. GmbH, Vechta, Germany). Each cage was equipped with one feeder and one watering device. The cages were located in separated house boxes with controlled temperature and humidity and with isolated watering systems. Air temperature was 20 °C. Starting from 10 days of age, the relative air humidity was 35 to 50%. The following lighting regime conditions were used: 20 hours of light (04:00 to 24:00 h) at 30–40 lux and 4 hours of darkness (24:00 to 04:00 h).

*Feeding Conditions*

Feeding of the chickens during the experiment period was carried out with crumbled mixed fodders using the compound feed PK1-2 G-3 (JSC Gatchinsky Feed Mill, Malye Kolpany, Russia) manufactured according to GOST R51851-2001. The birds received feed and water *ad libitum*.

The main ration based on the compound feed PK1-2 G-3 contained the following ingredients: wheat, sunflower meal, wheat bran, corn, limestone flour, limestone grains, yeast, dried shell, vegetable oil, defluorinated phosphate, premix P1-2 (0.5%), L-lysine monohydrochloride, salt, DL-methionine, Natuphos® (BASF SE, Ludwigshafen, Germany; 40 g/t), and Stafac® 110 (Phibro Animal Health Corporation, Teaneck, NJ, USA; 20 mg per 1 kg feed; 11% virginiamycin as an active ingredient). Metabolizable energy of the diet was 250 kcal per 100 g feed.

*Vaccination Scheme*

All birds from the day old and up to 95 days of age were vaccinated with vaccines listed in Table SI1-1.

**Table SI1-1.** Scheme of vaccination of laying hens used in the experiment.

| **Age at vaccination, days** | **Disease** | **Vaccine name** | **Vaccination technique** |
| --- | --- | --- | --- |
| 1 | Marek's disease (MD) | Rispens CVI 988 (Merial, Inc., Duluth, GA, USA) | subcutaneous injection in the upper third of the neck (0.2 ml) |
|  | MD + Infectious bursal disease (IBD, or Gumboro disease) | Vaxxitek HVT+IBD (Merial, USA) |  |
| 1 | IBD | Bioral Н-120 (Merial, USA) | spray |
|  | Newcastle disease (ND) | Avinew NEO (Merial, USA) |  |
| 12 | IBD | Gallivak IB-88 (Merial, USA) | spray |
| 20 | ND | Volvak ND LaSota MLV (Boehringer Ingelheim Mexico, Xaltocan, Mexico) | spray |
| 30 | Infectious laryngotracheitis (ILT) | Laryngo-Vac (Zoetis Inc., Parsippany, NJ, USA) | intraocular |
|  | ND | Gallimune ND (Merial, USA) | injection (0.3 ml) |
| 42 | Avian mycoplasmosis | MS-H (Bioproperties Pty Ltd, Ringwood, Australia) | intraocular |
| 43 | Avian metapneumovirus infection (aMPV) | Nemovak (Merial, USA) | spray |
| 50 | IBD | Volvak IB Mass MLV (Boehringer Ingelheim Mexico) | spray |
|  | ND | Avinew NEO (Merial, USA) | watering |
| 58 | ILT | Laryngo-Vac (Zoetis Inc.) | intraocular |
|  | Fowl pox | Avivac-Pox (SPE AVIVAC Ltd., Moscow, Russia) | intracutaneous |
| 65 | Avian encephalomyelitis | Avian encephalomyelitis vaccine (VNIIZZh, Vladimir, Russia) | watering |
| 72 | IBD | Gallivak IB-88 (Merial, USA) | spray |
| 80 | ND | Volvak ND LaSota MLV (Boehringer Ingelheim Mexico) | watering + spray |
| 95 | IBD + ND + Egg drop syndrome + aMPV | Gallimune 407 (ND+IB+EDS+ART) (Merial Italia S.p.a., Milan, Italy) | injection (0.3 ml) |
|  | IBD | Gallimune 793B (Merial, Lentilly, France) | injection (0.3 ml) |

*Experimental Design*

The four subgroups of laying hens were arranged by treatment and studied in the experiment as shown in Table SI1-2. Sampling was carried out at two time points, 1 day post inoculation (dpi) at 10 am and 7 dpi at 10 am.

**Table SI1-2.** Experimental design.

| **Subgroup** | **Treatment^1^** | **Diet** | **No. of animals** |
| --- | --- | --- | --- |
| S-I | Negative control (no challenge/phytobiotic) | Normal | 20 |
| S-II | SE challenge | Normal | 20 |
| S-III | Intebio intake | Normal + Intebio | 20 |
| S-IV | Intebio intake + SE challenge | Normal + Intebio | 20 |

^1^ SE, *Salmonella enterica* serovar Enteritidis; Intebio, phytobiotic (BIOTROF+ Ltd, Pushkin, St Petersburg, Russia).
