# Supplementary material for: Effects of Essential Oils-Based Supplement and Salmonella Infection on Gene Expression, Blood Parameters, Cecal Microbiome, and Egg Production in Laying Hens
Source: Animals (Basel). 2021 Feb 1;11(2):360. doi: 10.3390/ani11020360 (PMC7912222; doi:10.3390/ani11020360)
Supplement: Supplementary file 1 [file animals-11-00360-s001.zip › SuppInfo File S2.docx]

**Supporting Information File SI2**

*Effects of SE Challenge and Intebio Intake on Egg Production*

For the detailed analysis of influence SE challenge and phytobiotic supplement had on egg productivity traits in the subgroups, egg mass (*W*) was used, which is the product of two other traits, mean egg weight (*w*) and number of laid eggs (*N*), and reflects the productivity of the hens in a complex form. Thereby, eggs produced daily in each subgroup were taken into account. For convenience of perception, graphical dependences in Figure SI2-1 show a cycle of variability of *W* over the period of observations, including pairwise comparisons of individual subgroups.

A high variability of *W* during the observed period made it difficult to carry out the analysis and, therefore, a regression trend line was plotted for each pairwise comparison (Figure SI2-1b-e) as approximated by a linear functional dependence:

$W_{I}=-8.3979x+671.64$, (1)

$W_{II}=-7.8313x+594.91$, (2)

$W_{III}=-3.1974x+612.07$, (3)

$W_{IV}=-5.2271x+576.11$, (4)

where *W_I_*, *W_II_*, *W_III_* и *W_IV_* are mass of eggs laid in one day in S-I to S-IV, respectively; *x* is the appropriate day of the observation period equal to 1 to 27.

Comparison of dependencies in S-I and S-II (Figure SI2-1b) suggests a steady decline (slope) in their egg mass over the entire period. Comparison of S-I and S-III (Figure SI2-1c) shows that starting from about the middle of the observation period, S-III begins to dominate over S-I. When comparing S-I and S-IV (Figure SI2-1d), it can be noted that S-IV is gradually aligned with the control S-I, while S-III outgoes S-IV (Figure SI2-1e).

Since the *W* data reflects a general tendency of a lowering post-peak egg production during the observation period, the obtained equations (1) to (4) allow us to calculate a decrease rate of these functions. To calculate the decline rate of egg production in S-I and S-II, the values of *x* = 1 and 27 were substituted into the corresponding equations (1) and (2) for days of the beginning and end of observation, and the decrease rate of egg mass, *ν* (g/day), was calculated by the following formula:

$v=\frac{W_{1}-W_{27}}{27}$. (5)

Then,

$v_{I}=\frac{663.24-444.9}{27}=8.09$, (6)

$v_{II}=\frac{587.08-383.46}{27}=7.54$. (7)

The obtained values of *ν* are very close to each other, which is indicative of a similar trend in the post-peak egg production recession in laying hens in both S-I and S-II.

**(a)**

**(b)** (**c)**

**(d)** (**e)**

**Figure SI2-1.** Graphical representation of egg mass variation dependences in all subgroups over 27-day egg-laying period (a) and pairwise comparative analysis between Subgroups I and II (b), I and III (c), I and IV (d), and II and IV (e).

Similar calculations were performed for the egg production recession rate in the two other subgroups. Data on the decrease rate using the appropriate egg mass and *ν* functions is summarized in Table SI2-1 showing a rather slower decline rate of egg productivity in S-III and S-IV as compared with S-I and S-II.

**Table SI2-1.** Decline rate of functions reflecting mass of eggs laid by a subgroup of chickens per 1 day.

| **Subgroup** | **Treatment** | **Decrease rate of egg production (*ν*; g/day)** |
| --- | --- | --- |
| S-I | Negative control (no challenge/phytobiotic) | 8.09 |
| S-II | SE challenge | 7.54 |
| S-III | Intebio intake | 3.08 |
| S-IV | Intebio intake + SE challenge | 5.03 |
