# Supplementary material for: Effects of Essential Oils-Based Supplement and Salmonella Infection on Gene Expression, Blood Parameters, Cecal Microbiome, and Egg Production in Laying Hens
Source: Animals (Basel). 2021 Feb 1;11(2):360. doi: 10.3390/ani11020360 (PMC7912222; doi:10.3390/ani11020360)
Supplement: Supplementary file 1 [file animals-11-00360-s001.zip › SuppInfo Table S1.docx]

**Table S1.** Gene specific primers for assessing the expression of eight chicken genes.

| **Gene symbol** | **Gene name (synonym)** | **Function^1^** | **GenBank Accession No.** | **Primer sequence (5’-3’)^2^** | **PCR product size (bp)** | **Reference** |
| --- | --- | --- | --- | --- | --- | --- |
| *AvBD10* | avian β-defensin 10 (gallinacin-10) | antimicrobial peptide | CR388516 | F: GCTCTTCGCTGTTCTCCTCT  R: CCCAGAGATGGTGAAGGTG | 67 | [1] |
| *IL6* | interleukin 6 (cytokine) | chemokine, response to inflammation and infection | AJ309540 | F: AGGACGAGATGTGCAAGAAGTTC  R: TTGGGCAGGTTGAGGTTGTT | 78 | [2] |
| *IL8L1* | interleukin 8-like 1 (*CXCLi2*, or CXC chemokine K60) | chemokine, inflammatory activity | Y14971 | F: AGACTCATTCCAAGTTCATCCA  R: TTTGTTCTTTGCTTTAGGATGC | 216 | [2] |
| *SLC5A1* | solute carrier family 5 member 1 (sodium/glucose cotransporter member 1, *SGLT1*) | glucose and Na^+^ transmembrane transport, intestinal hexose absorption | XM_415247 | F: AGCATTTCAGCATGGTGTGTCTTC  R: GATGCTCCTATCTCAGGGCAGTTC | 113 | [3] |
| *CA2* | carbonic anhydrase 2 | carbonate dehydratase activity, HCO_3_^−^ and CO_2_ transport, eggshell calcification | NM_205317 | F: ATCGTCAACAACGGGCACTCCTTC  R: TGCACCAACCTGTAGACTCCATCC | 156 | [4] |
| *CALB1* | calbindin 1 | calcium ion binding, calcium transport | NM_205513 | F: CAGGGTGTCAAAATGTGTGC  R: GCCAGTTCTGCTCGGTAAAG | 102 | [4] |
| *RARRES1* | retinoic acid receptor responder 1 (ovocalyxin-32, *OCX32*) | negative regulation of endopeptidase activity; protein that makes up the eggshell matrix | AB547158 | F: AGAAGAGGACCACAGATTTTACGAA  R: GATGGTCATGGGCAATATTTCC | 119 | [5] |
| *BPIFB3* | BPI fold containing family B member 3 (ovocalyxin-36, *OCX36*) | lipid binding, innate immune response; protein that makes up the eggshell matrix | XM_025142226 | F: TTGGAATGGTCGTCTTCTGTGG  R: CGGTCTGAATGATGGCATCG | 126 | [6] |

^1^ Information derived from NCBI databases. ^2^ Forward (F) and reverse (R) primers were designed, given their annealing temperature around 59 °C.

References

1. Li, X.; Swaggerty, C.L.; Kogut, M.H.; Chiang, H.I.; Wang, Y.; Genovese, K.J.; He, H.; Zhou, H. Gene expression profiling of the local cecal response of genetic chicken lines that differ in their susceptibility to *Campylobacter jejuni* colonization. *PLoS One* **2010**, *5*, art. e11827.
2. Chiang H.I.; Swaggerty, C.L.; Kogut, M.H.; Dowd, S.E.; Li, X.; Pevzner, I.Y.; Zhou, H. Gene expression profiling in chicken heterophils with *Salmonella* enteritidis stimulation using a chicken 44 K Agilent microarray. *BMC Genomics* **2008**, *9*, art. 526.
3. Feng, Y.; Yang, X.J.; Wang, Y.B.; Li, W.L.; Liu, Y.; Yin, R.Q.; Yao, J.H. Effects of immune stress on performance parameters, intestinal enzyme activity and mRNA expression of intestinal transporters in broiler chickens. *Asian-Australas. J. Anim. Sci.* **2012**, *25*, 701–707.
4. Jonchère, V.; Brionne A.; Gautron, J.; Nys Y. Identification of uterine ion transporters for mineralisation precursors of the avian eggshell. *BMC Physiol.* **2012**, *12*, art. 10.
5. Yang, K.T.; Lin, C.Y.; Liou, J.S.; Fan, Y.H.; Chiou, S.H.; Huang, C.W.; Wu, C.P.; Lin, E.C.; Chen, C.F.; Lee, Y.P.; Lee, W.C.; Ding, S.T.; Cheng, W.T.; Huang, M.C. Differentially expressed transcripts in shell glands from low and high egg production strains of chickens using cDNA microarrays. *Anim. Reprod. Sci.* **2007**, *101*, 113–124.
6. Gautron, J.; Murayama, E.; Vignal, A.; Morisson, M.; McKee, M.D.; Réhault, S.; Labas, V.; Belghazi, M.; Vidal, M.L.; Nys, Y.; Hincke, M.T. Cloning of ovocalyxin-36, a novel chicken eggshell protein related to lipopolysaccharide-binding proteins, bactericidal permeability-increasing proteins, and Plunc family proteins. *J. Biol. Chem.* **2007**, *282*, 5273–5286.
