# Supplementary material for: Effects of Essential Oils-Based Supplement and Salmonella Infection on Gene Expression, Blood Parameters, Cecal Microbiome, and Egg Production in Laying Hens
Source: Animals (Basel). 2021 Feb 1;11(2):360. doi: 10.3390/ani11020360 (PMC7912222; doi:10.3390/ani11020360)
Supplement: Supplementary file 1 [file animals-11-00360-s001.zip › SuppInfo Table S2.docx]

**Table S2.** Blood biochemical/immunological parameters in laying hens at 1 dpi.

| **Blood parameters** | | **Subgroups^1^** | | | | **Reference range^2^** |
| --- | --- | --- | --- | --- | --- | --- |
|  |  | **S-I** | **S-II** | **S-III** | **S-IV** |  |
| Total protein, g/l | | 60.0 ± 3.0^a^ | 57.1 ± 3.6^a^ | 59.2 ± 3.1^a^ | 53.8 ± 3.1^a^ | 43–60 |
| Albumin, g/l | | 15.8 ± 0.7^a^ | 11.0 ± 0.45^b^ | 15.4 ± 0.78^a^ | 11.3 ± 0.45^b^ | 1.96–21.7 |
| Globulins, g/l | | 44.2 ± 2.2^a^ | 46.1 ± 2.1^a^ | 43.8 ± 2.4^a^ | 42.5 ± 1.7^a^ | 2.5–20.3 |
| Albumin, % | | 26.7 ± 1.4^a^ | 20.1 ± 0.8^b^ | 26.7 ± 1.2^a^ | 21.7 ± 1.5^b^ | 31.4–35.1 |
| Globulins, % | | 73.3 ± 3.8^a^ | 79.9 ± 2.5^b^ | 73.3 ± 3.7^a^ | 78.9 ± 4.7^b^ | 10.9–37.1 |
| Urea, mmol/l | | 2.9 ± 0.17^a^ | 1.3 ± 0.04^b^ | 1.9 ± 0.07^a,b^ | 4.1 ± 0.9^c^ | 0.31–3.4 |
| Urea nitrogen, mmol/l | | 1.4 ± 0.07^a^ | 0.61 ± 0.01^b^ | 0.9 ± 0.05^c^ | 1.9 ± 0.07^d^ | N/A |
| Creatinine, μmol/l | | 41.6 ± 1.9^a,b^ | 40.4 ± 1.7^a^ | 48.0 ± 2.1^b^ | 53.3 ± 1.8^c^ | 69–524 |
| Alanine aminotransferase, IU/l | | 14.3 ± 0.69^b^ | 6.7 ± 0.32^a^ | 7.6 ± 0.32^a^ | 15.4 ± 0.45^b^ | 1.67–9.94 |
| Aspartate aminotransferase, IU/l | | 331.6 ± 14.2^b^ | 297.1 ± 12.4^c^ | 200.4 ± 8.4^a^ | 228.8 ± 8.7^a^ | 107–481 |
| Alkaline phosphatase, IU/l | | 244.4 ± 9.9^a^ | 226.1 ± 10.3^a^ | 388.3 ± 15.6^b^ | 312.3 ± 11.3^c^ | 400–1100 |
| Alpha amylase, IU/l | | 527.8 ± 21.7^a^ | 972.5 ± 35.9^c^ | 599.7 ± 25.9^a,b^ | 675.5 ± 55.9^b^ | N/A |
| Glucose, mmol/l | | 13.3 ± 0.6^a^ | 12.4 ± 0.54^a^ | 13.0 ± 0.65^a^ | 13.6 ± 2.3^a^ | 22.3–39.0 |
| Cholesterol, total, mmol/l | | 1.8 ± 0.07^a^ | 1.1 ± 0.04^b^ | 3.3 ± 0.12^c^ | 1.3 ± 0.05^d^ | 2.8–5.2 |
| Calcium, total, mmol/l | | 4.2 ± 0.18^a^ | 3.7 ± 0.14^b,c^ | 4.1 ± 0.2^a,b^ | 3.3 ± 0.14^c^ | 2.0–5.0 |
| Phosphorus, mmol/l | | 2.3 ± 0.11^b^ | 1.7 ± 0.06^a^ | 2.5 ± 0.08^c^ | 1.6 ± 0.07^a^ | 0.64–1.45 |
| Bilirubin, μmol/l | | 0.62 ± 0.02^b^ | 0.49 ± 0.01^c^ | 0.83 ± 0.02^a^ | 0.84 ± 0.05^a^ | N/A |
| Uric acid, μmol/l | | 202.5 ± 9.4^a^ | 143.2 ± 6.8^b^ | 191.3 ± 9.9^a^ | 102.2 ± 10.4^c^ | 119–892 |
| Lysozyme activity, % | | 9.3 ± 0.3^b^ | 12.8 ± 0.55^a,c^ | 13.7 ± 0.55^a^ | 10.2 ± 2.1^b,c^ | N/A |
| Bactericidal activity, % | | 55.3 ± 2.5^a,b^ | 46.6 ± 1.9^a^ | 50.7 ± 1.8^a,b^ | 59.4 ± 6.5^b^ | N/A |
| Beta-lysine activity, % | | 70.9 ± 3.2^b^ | 62.7 ± 2.5^a,c^ | 57.9 ± 2.1^a^ | 68.7 ± 3.9^b,c^ | N/A |
| Immunoglobulin activity | IgA | 1.2 ± 0.03^b^ | 0.92 ± 0.08^c^ | 1.4 ± 0.04^a^ | 1.5 ± 0.08^a^ | N/A |
|  | IgM | 1.2 ± 0.01^a^ | 0.34 ± 0.02^b^ | 1.3 ± 0.05^a^ | 0.35 ± 0.12^b^ | N/A |
|  | IgG1 | 13.8 ± 0.54^a^ | 2.1 ± 0.1^b^ | 17.3 ± 0.04^c^ | 6.8 ± 0.35^d^ | N/A |
|  | IgG2 | 16.5 ± 0.5^a^ | 11.9 ± 0.7^b^ | 18.3 ± 1.3^a^ | 9.8 ± 0.53^b^ | N/A |

^1^ Subgroups: S-I (negative control), S-II (SE challenge), S-III (Intebio intake), S-IV (Intebio intake + SE challenge).

^2^ As compiled in [1].

^a-d^ Data within each raw (blood parameter) with no common letters differed significantly (at *p* ≤ 0.05).

**Reference**

1. Nasonov, I.V.; Buyko, N.V.; Lizun, R.P.; Volykhina, V.E.; Zakharik, N.V.; Yakubovsky, S.M. [*Guidelines for Hematological and Biochemical Studies in Chickens of Modern Crosses*]. Ministry of Agriculture and Food of the Republic of Belarus, RUE S. Vyshlesky Institute of Experimental Veterinary Medicine of the National Academy of Sciences of Belarus: Minsk, Belarus, 2014; 32 p.
