# Supplementary material for: Effects of Essential Oils-Based Supplement and Salmonella Infection on Gene Expression, Blood Parameters, Cecal Microbiome, and Egg Production in Laying Hens
Source: Animals (Basel). 2021 Feb 1;11(2):360. doi: 10.3390/ani11020360 (PMC7912222; doi:10.3390/ani11020360)
Supplement: Supplementary file 1 [file animals-11-00360-s001.zip › SuppInfo Table S3.docx]

**Table S3.** Blood biochemical/immunological parameters in laying hens at 7 dpi.

| **Blood parameters** | | **Subgroups^1^** | | | | **Reference range^2^** |
| --- | --- | --- | --- | --- | --- | --- |
|  |  | **S-I** | **S-II** | **S-III** | **S-IV** |  |
| Total protein, g/l | | 64.1 ± 3.1^a^ | 59.7 ± 2.5^a^ | 64.9 ± 4.2^a^ | 57.8 ± 5.3^a^ | 43–60 |
| Albumin, g/l | | 14.1 ± 0.5^b^ | 11.9 ± 0.45^a^ | 12.8 ± 0.55^a,b^ | 14.1 ± 0.62^b^ | 1.96–21.7 |
| Globulins, g/l | | 49.9 ± 2.9^a^ | 47.8 ± 2.1^a^ | 52.1 ± 2.1^a^ | 43.7 ± 6.3^a^ | 2.5–20.3 |
| Albumin, % | | 22.0 ± 1.5^a,b^ | 19.8 ± 0.88^a^ | 19.7 ± 2.4^a^ | 24.5 ± 1.0^b^ | 31.4–35.1 |
| Globulins, % | | 78.0 ± 3.4^a,b^ | 80.2 ± 3.3^a^ | 80.3 ± 3.5^a^ | 75.5 ± 3.5^b^ | 10.9–37.1 |
| Urea, mmol/l | | 3.4 ± 0.15^a^ | 4.9 ± 0.57^b^ | 3.5 ± 0.15^a^ | 3.0 ± 0.12^a^ | 0.31–3.4 |
| Urea nitrogen, mmol/l | | 1.6 ± 0.07^a,b^ | 2.3 ± 0.15^c^ | 1.7 ± 0.07^a^ | 1.4 ± 0.04^b^ | N/A |
| Creatinine, μmol/l | | 44.7 ± 2.2^a^ | 54.0 ± 1.8^b^ | 46.3 ± 2.8^a^ | 43.8 ± 2.6^a^ | 69–524 |
| Alanine aminotransferase, IU/l | | 5.8 ± 0.21^a^ | 9.5 ± 0.3^b^ | 7.6 ± 0.92^a,b^ | 20.6 ± 1.5^c^ | 1.67–9.94 |
| Aspartate aminotransferase, IU/l | | 217.2 ± 10.9^a^ | 276.6 ± 11.1^b^ | 178.2 ± 9.4^c^ | 219.9 ± 10.6^a^ | 107–481 |
| Alkaline phosphatase, IU/l | | 330.7 ± 12.5^b^ | 268.3 ± 13.9^a,c^ | 252.5 ± 12.6^a^ | 291.8 ± 19.7^b,c^ | 400–1100 |
| Alpha amylase, IU/l | | 469.4 ± 25.6^b^ | 404.3 ± 15.6^a^ | 487.4 ± 29.8^b^ | 447.0 ± 22.5^a,b^ | N/A |
| Glucose, mmol/l | | 12.2 ± 0.87^b^ | 10.1 ± 0.4^a^ | 10.3 ± 0.44^a^ | 10.3 ± 0.5^a^ | 22.3–39.0 |
| Cholesterol, total, mmol/l | | 2.8 ± 0.15^a^ | 2.6 ± 0.1^a^ | 2.7 ± 0.11^a^ | 3.3 ± 0.2^b^ | 2.8–5.2 |
| Calcium, total, mmol/l | | 4.7 ± 0.25^a^ | 4.5 ± 0.12^a^ | 4.8 ± 0.22^a^ | 4.8 ± 0.26^a^ | 2.0–5.0 |
| Phosphorus, mmol/l | | 3.3 ± 0.14^a^ | 2.3 ± 0.11^b^ | 1.9 ± 0.07^c^ | 3.4 ± 0.17^a,b^ | 0.64–1.45 |
| Bilirubin, μmol/l | | 0.68 ± 0.06^a^ | 0.71 ± 0.02^a^ | 0.49 ± 0.01^b^ | 1.27 ± 0.12^c^ | N/A |
| Uric acid, μmol/l | | 259.8 ± 16.7^b^ | 119.9 ± 4.7^a^ | 185.6 ± 15.3^c^ | 136.7 ± 6.5^a^ | 119–892 |
| Lysozyme activity, % | | 10.5 ± 0.53^b^ | 15.5 ± 0.75^a^ | 15.3 ± 0.55^a^ | 14.0 ± 0.45^a^ | N/A |
| Bactericidal activity, % | | 58.1 ± 2.3^a,b^ | 37.9 ± 1.4^c^ | 53.0 ± 2.98^a^ | 63.1 ± 3.1^b^ | N/A |
| Beta-lysine activity, % | | 65.1 ± 3.6^a^ | 56.4 ± 2.2^b^ | 62.1 ± 3.5^a,b^ | 75.2 ± 3.2^c^ | N/A |
| Immunoglobulin activity | IgA | 2.2 ± 0.1^a^ | 1.9 ± 0.07^a,b^ | 1.5 ± 0.14^c^ | 1.6 ± 0.15^b,c^ | N/A |
|  | IgM | 0.8 ± 0.03^a^ | 1.8 ± 0.05^b^ | 1.5 ± 0.07^c^ | 2.7 ± 0.18^d^ | N/A |
|  | IgG1 | 11.0 ± 0.59^a^ | 8.5 ± 0.77^b^ | 6.0 ± 0.32^c^ | 12.5 ± 0.78^a^ | N/A |
|  | IgG2 | 5.1 ± 0.26^a^ | 5.8 ± 0.31^b^ | 4.9 ± 0.21^a^ | 4.7 ± 0.22^a^ | N/A |

^1^ Subgroups: S-I (negative control), S-II (SE challenge), S-III (Intebio intake), S-IV (Intebio intake + SE challenge).

^2^ As compiled in [1].

^a-d^ Data within each raw (blood parameter) with no common letters differed significantly (at *p* ≤ 0.05).

**Reference**

1. Nasonov, I.V.; Buyko, N.V.; Lizun, R.P.; Volykhina, V.E.; Zakharik, N.V.; Yakubovsky, S.M. [*Guidelines for Hematological and Biochemical Studies in Chickens of Modern Crosses*]. Ministry of Agriculture and Food of the Republic of Belarus, RUE S. Vyshlesky Institute of Experimental Veterinary Medicine of the National Academy of Sciences of Belarus: Minsk, Belarus, 2014; 32 p.
